# A stress-sensing circuit signals to the central pacemaker to reprogram circadian rhythms

**DOI:** 10.1126/sciadv.adr7960

**Published:** 2025-06-20

**Authors:** Maria E. Yurgel, Claire Gao, John J. O’Malley, Qijun Tang, Noam Yanay, Alison R. Bashford, Jesse J. Zhan, Andrew Lutas, Michael J. Krashes, Haiqing Zhao, Mario A. Penzo, Samer Hattar

**Affiliations:** ^1^ Section on Light and Circadian Rhythms (SLCR), National Institute of Mental Health, National Institutes of Health (NIMH), Bethesda, MD 20892, USA.; ^2^ Section on the Neural Circuits of Emotion and Motivation, NIMH, Bethesda, MD 20892, USA.; ^3^ Diabetes, Endocrinology, and Obesity Branch, National Institute of Diabetes and Digestive and Kidney Diseases, National Institutes of Health, Bethesda, MD 20892, USA.; ^4^ Department of Biology, Johns Hopkins University, Baltimore, MD 21218, USA.

## Abstract

The circadian system provides a temporal framework for animals to anticipate environmental events, including threats. However, the effects of stressors on the circadian system remain poorly understood. Here, we demonstrate that, in mice, stressors shift the phase of the central pacemaker, housed in the suprachiasmatic nucleus (SCN), through glutamatergic inputs from the anterior paraventricular nucleus of the thalamus (aPVT). Unlike light, which can phase delay or advance the central pacemaker, stressors consistently induce delays, effects attenuated by inhibiting aPVT neurons. Stressors robustly activate AVP-expressing neurons within the SCN and are associated with inhibition of VIP-expressing neurons, whereas light strongly activates VIP-expressing neurons with minimal effects on AVP-expressing neurons. Pairing stressors with light reveals distinct time-dependent interactions, enhancing phase delays at early night but abolishing phase advances at late night. Our findings uncover distinct SCN microcircuits that differentially encode light and stressors, providing insights into how environmental cues modulate circadian timing.

## Teaser

Stress, via the paraventricular thalamus, modifies the central pacemaker and interacts with light to influence circadian outputs.

## INTRODUCTION

The brain’s capacity to detect and anticipate environmental threats is essential for orchestrating adaptive behaviors. The circadian system provides a temporal niche that enables animals to predict and respond effectively to such challenges (*1*). Consequently, aversive stimuli may significantly influence time-dependent behavioral patterns. In humans, stress-related disorders such as post-traumatic stress disorder are linked to disturbances in both sleep and circadian rhythms (*2*–*4*), underscoring the interplay between stress and circadian regulation. However, relatively few studies have explored the impact of aversive stimuli on circadian rhythms, and their mixed findings have limited our understanding of the neural mechanisms involved (*5*–*12*).

The suprachiasmatic nucleus (SCN) of the hypothalamus functions as a central pacemaker, integrating both photic (light) and non-photic (such as exercise and arousal) cues to construct a temporal representation of the environment (*13*). A hallmark of circadian rhythms is their differential response to environmental inputs depending on time of day, a principle that underlies the derivation of phase response curve (*14*–*16*). For instance, photic stimulation at the onset of a rodent’s active period typically shifts circadian rhythms to a later phase (phase delay), whereas stimulation at the end of the active period triggers an earlier phase (phase advances) (*17*–*21*). By contrast, non-photic signals induce phase advances during the inactive period (*22*–*25*). This time-dependent variability enables the circadian clock to integrate multiple environmental cues and maintain coherent rhythmic function.

Studies investigating the effects of aversive stimuli on the central circadian pacemaker have produced varied findings, influenced by both the type of stressor and the animal model used (*5*–*12*). For instance, exposing mice to 3 consecutive days of restraint stress, whether at the start of the day or night, did not alter SCN expression of the canonical clock gene, *Per2* (*8*). In golden hamsters, however, immobilization at the beginning of the night delayed the central pacemaker (*12*), while footshock administered midday advanced its phase (*5*). Notably, recent work showed that cyclic fear, induced by footshocks, shifts locomotor activity and feeding rhythms in both rats and mice to align with the safe phase of the 24-hour shock cycle (*6*, *7*). Despite these insights, the neural pathways that relay aversive signals to the SCN remain to be elucidated.

The paraventricular thalamus (PVT) is a strong putative candidate region for the interaction between stressors and the circadian system. Beyond its established roles in cognitive and emotional processes through wide-ranging projections (*26*–*28*), the PVT critically modulates stress by coordinating both neuroendocrine and behavioral adaptations (*29*–*33*). Unlike most thalamic nuclei, it is reciprocally connected with the hypothalamus, enabling it to broadcast stress-related signals and to receive internal state information (*26*). Notably, emerging evidence highlights a bidirectional relationship between the PVT and the SCN (*34*–*39*). This unique connectivity positions the PVT to integrate stress signals with circadian timing, as illustrated by its involvement in anticipatory feeding behavior (*40*). Thus, the PVT’s anatomical and functional properties support a model, in which it serves as a key conduit for conveying aversive and homeostatic cues to the SCN, providing a mechanistic basis for how stress can shape circadian rhythms.

In the work presented here, we examine how acute stressors affect the central circadian pacemaker and delineate the underlying neural mechanisms. Our data show that aversive inputs, routed via the anterior PVT (aPVT), reprogram the SCN in ways that differ markedly from photic stimulation. Notably, pairing light with stressors enhances phase delays at the beginning of the night but abolishes light-induced phase advances at its end, illustrating how the SCN integrates multiple environmental signals. These findings reveal specific circuits through which stress influences circadian control and offer a previously unidentified framework for investigating the relationship between stress systems and circadian rhythms in anxiety-related disorders.

## RESULTS

### Stressors affect locomotor activity rhythms differently than light during the mouse’s active period

**Fig. 1.**
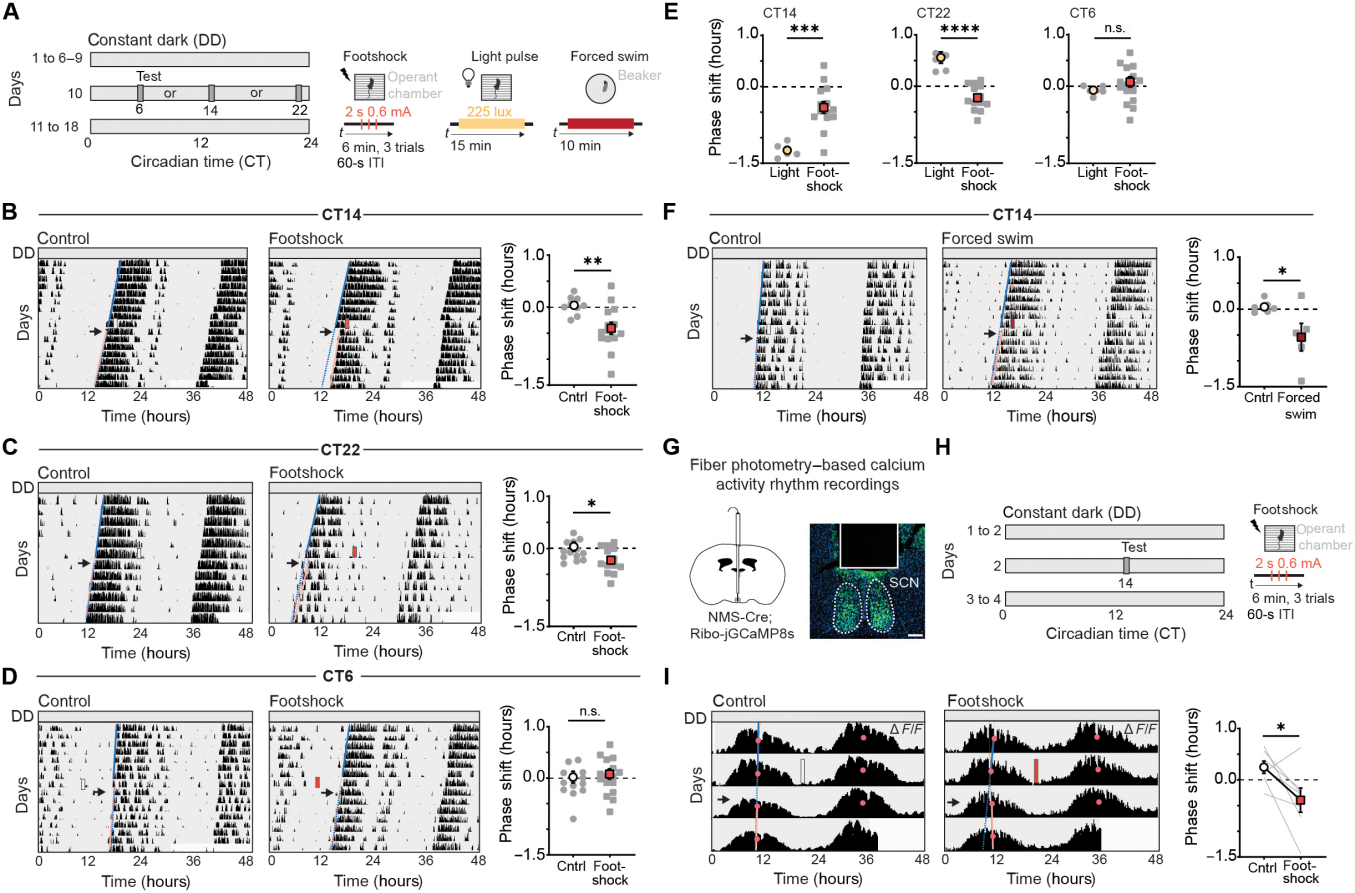
Stressors phase shift circadian rhythms differently than light. (**A**) Schematic of experimental paradigm. (**B** to **D**) Wheel running activity profiles of control mice (white rectangle), and mice subjected to footshock session (red rectangle) at CT14 (B), CT22 (C), and CT6 (D). Rectangles represent day and time of test. Blue solid line depicts activity onset before test; blue dotted line depicts expected activity onsets, and pink regression line depicts shift in activity onset posttest. Black arrow indicated the first day posttest. Scatter plots represent quantification of the phase shifts (hours) in controls and mice subjected to footshocks at CT14 (B), CT22 (C), and CT6 (D). Data are means ± SEM. CT14, *P = 0.01 (*n* = 9 to 14 mice). CT22, *P = 0.03 (*n* = 13 to 14 mice). CT6, nonsignificant (n.s., *n* = 15 to 17 mice). Student’s *t* test. (**E**) Comparison between phase shift (hours) in response to light (*n* = 6 mice) and footshock (*n* = 13 to 17) at CT6, CT14, and CT22. Data are means ± SEM. ***P = 0.002; ****P < 0.0001, Student’s *t* test. (**F**) Running-wheel activity profiles of control mice (white), and mice subjected to a forced swim session (dark red) and quantification of the phase shifts (hours) in controls and mice subjected to forced swim at CT14. Data are means ± SEM (*n* = 5 to 6 mice). *P = 0.04, Student’s *t* test. (**G**) Schematic of fiber implantation in the SCN of NMS-Cre;GCaMP8s mice and representative image of GCaMP8s expression and fiber placement. Scale bar, 100 μm. (**H**) Schematic of experimental paradigm. (**I**) Representative calcium activity profiles in response to a sham and a footshock session. Acrophase (pink circles) was used to calculate phase shifts. Quantification of the phase shifts (hours) in controls, and mice subjected to footshocks. Data are means ± SEM (*n* = 7 mice). *P = 0.04, two-tailed paired-sample *t* test. Cntrl, control; ITI, intertrial interval.

To investigate whether stressors affect the SCN of the hypothalamus, we subjected wild-type (WT) C57BL/6J mice to footshocks at different times of the day (Fig. 1A). The SCN, known as the central pacemaker, is essential for synchronizing clocks throughout the brain and peripheral tissues with external environmental cues (*13*). We chose to compare the effects of footshocks with those of light exposure because light is the primary synchronizing cue for the SCN and has been extensively characterized in rodents (Fig. 1A) (*13*). Light’s predictable, cyclic nature and controllability make it an ideal benchmark for elucidating whether stress-induced changes in timed behaviors would mimic or differ from light.

WT mice were kept in constant darkness (DD) for 6 to 7 days, and, then, footshocks or light pulses were administered at circadian time 14 (CT14) and CT22, corresponding to the beginning and end of the active period (nighttime), and at CT6, corresponding to the middle of the inactive period (daytime). Locomotor activity rhythms were recorded for the next 6 to 7 days, and phase shifts were quantified on the basis of the onset of activity following footshock or light sessions (Fig. 1A). Control mice were placed in the operant chamber without receiving any stimulation.

Footshocks resulted in significant phase delays of ~0.4 hours at CT14 (Fig. 1B and fig. S1, A, H, and I). This phase-delaying effect is similar to that of light (~1.2 hours) but weaker (Fig. 1E and fig. S1, E and F). At CT22, footshocks caused significant phase delays (~0.2 hours), albeit milder than at CT14 (Fig. 1C and fig. S1, H, and I). In contrast, light induced phase advances (~0.5 hours) at CT22 (Fig. 1E and fig. S1, E, and F). At CT6, neither light nor footshocks altered the phase of the locomotor activity rhythms (Fig. 1, D and E; and fig. S1, A, E, F, H, and I). Control mice exhibited little phase alterations at all circadian times (Fig. 1, B to D, and fig. S1H). Of note, a two-way analysis of variance (ANOVA) comparing phase shifts in response to footshock across circadian times revealed a significant difference from controls only at CT14 (fig. S1A). The free-running period before and after the stimulus remained unaffected in both control and footshock-treated mice at all CT times (fig. S1, B to D). These data show that footshock stress can phase shift locomotor activity rhythms. The magnitude and direction of the response to stressors at different times of the day are distinct from those of light, suggesting that the SCN may differentially encode photic and non-photic information.

To determine whether the results observed in response to footshocks were generalizable to other stressors we subjected mice to forced swim stress, a type of stressor that lacks a nociceptive component, for 10 min (*41*) at CT14, the time point at which we observed the largest effects of footshocks on circadian phase (Fig. 1A). Mice subjected to forced swim stress showed phase delays that were comparable to those induced by footshocks (Fig. 1F and fig. S1, H and I), further supporting the notion that stressors phase delay locomotor activity rhythms.

To test whether stressors reprogram the central pacemaker or affect locomotor activity rhythms independent of the SCN (*6*), we used long-term fiber photometry to record calcium dynamics within the SCN across the daily cycle (Fig. 1G). A cannula was implanted above the SCN of Neuromedin S (NMS)-Cre mice expressing soma-targeted genetically encoded calcium indicator, 8 sensitive (GCaMP8s). NMS is broadly expressed in the SCN (SCN^NMS+^), encompassing ~40% of SCN neurons (*42*). After confirming the reliability of daily calcium rhythm recordings (fig. S1G), mice were exposed to a footshock session at CT14 in constant darkness, and calcium activity was continuously monitored (Fig. 1H). Phase shifts in calcium rhythms were quantified on the basis of the peak point of the calcium cycle, known as acrophase. Footshock-exposed mice exhibit a significant phase delay (~0.4 hours) compared to controls (Fig. 1I), matching the shift observed in locomotor activity rhythms (Fig. 1B). These findings demonstrate that stressors cause phase shifts in locomotor activity by reprogramming the central pacemaker.

**Fig. 2.**
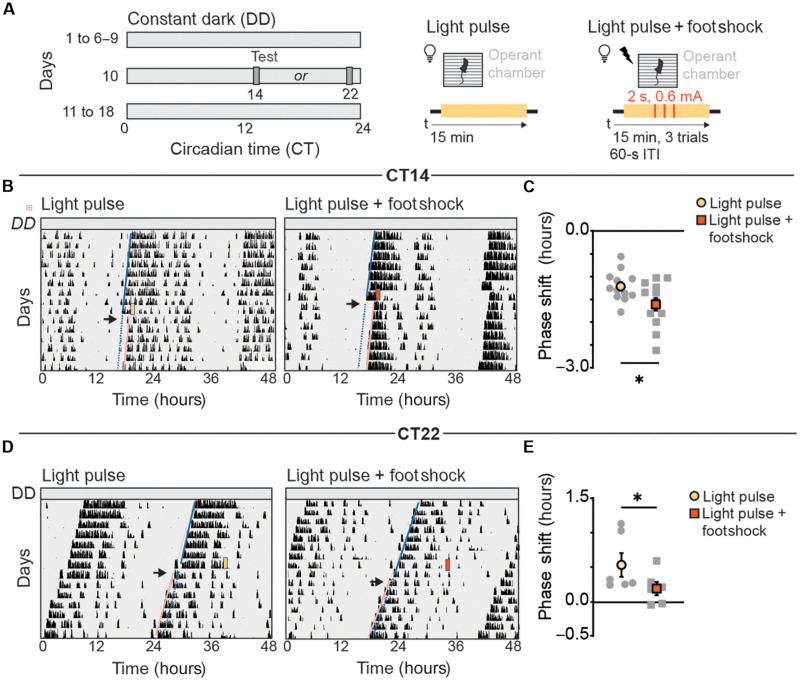
Pairing light and stressors results in additive phase delays at CT14 and reduces light-induced phase advances at CT22. (**A**) Schematic of experimental paradigm; mice were placed in constant dark (DD) for 6 to 9 days and then subjected to a light pulse (middle) or light pulse and footshock session (right) at CT14 and CT22. Wheel running activity was recorded for an additional 7 days. (**B** and **D**) Running-wheel activity profiles of mice exposed to a light pulse alone (yellow) or light pulse paired with footshocks (orange) at CT14 (B) and CT22 (D). Blue solid line depicts activity onset before test, blue dotted line depicts expected activity onsets, and pink line depicts shift in activity onset posttest. Black arrow indicated the first day posttest. (**C** and **E**) Quantification of the phase shifts (hours) in light only (yellow) or light paired with footshock (orange) groups at CT14 (C) and CT22 (E). Data are means ± SEM. CT14 (*n* = 13 mice), *P = 0.028, Student’s *t* test. CT22 (*n* = 6 mice), *P = 0.035, two-tailed paired *t* test. ITI, intertrial interval.

The circadian system integrates photic and non-photic cues to organize metabolic, physiological, and behavioral rhythms (*43*–*48*). Therefore, we explored the interactions between stressors and light by exposing mice to a moderate light pulse paired with footshock at CT14 (130 lux) and CT22 (225 lux) (Fig. 2A). These time points were selected because both light and footshocks influence the phase of the central pacemaker: Light induces phase delays at CT14 and advances at CT22, whereas shocks exclusively cause phase delays (Fig. 1E and fig. S1, E and F).

The 130 lux light pulse induced ~1.2-hour phase delays at CT14 (Fig. 2, B and C), while the 225 lux light pulse produced ~0.5-hour phase advances at CT22 (Fig. 2, D and E). Pairing footshocks with light at CT14 enhanced the phase delay to ~1.6 hours (Fig. 2, B and C). In contrast, at CT22, combining light and footshocks reduced the light-induced phase advances to ~0.2 hours (Fig. 2, D and E). A two-way ANOVA comparing the effects of light paired with footshocks across circadian times revealed that the ~0.2-hour phase advance observed at CT22 was not significantly different from controls kept in darkness (fig. S2A). Thus, at CT14, pairing light and stressors results in enhanced phase delays, whereas, at CT22, the combination eliminates phase advances. Together, these data highlight the strength of aversive experiences in attenuating the phase-advancing effects of light and support the notion that the SCN balances photic and non-photic cues to adjust its circadian response.

### SCN neurons differentially encode light and aversive information

**Fig. 3.**
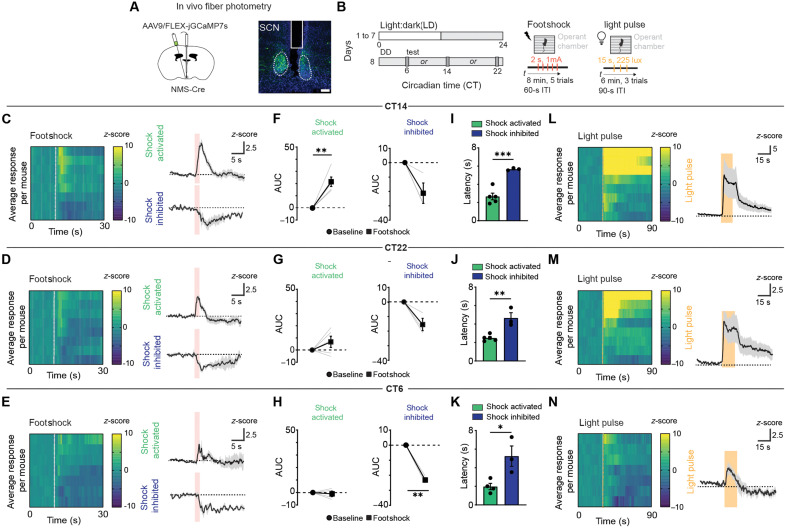
SCN^NMS*+*^ neurons are differentially responsive to aversive stimuli across CT times. (**A**) Schematic of the stereotaxic injection to selectively target expression of GCaMP7s to NMS-expressing SCN neurons (left). Representative image of GCaMP7s expression in the SCN and fiber placement (right). Scale bar, 100 μm. (**B**) Schematic of experimental paradigm. (**C** to **E**) Heatmaps of average calcium responses to footshocks (five trials) per mouse and average responses for mice displaying increases in fluorescence levels (shock activated; green) and decreases in fluorescence levels (shock inhibited; blue) at CT14 (C), CT22 (D), or CT6 (E). (**F** to **H**) Quantification of GCaMP7s responses to footshocks. Area under the curve (AUC) comparison between baseline (0 to 10 s; circle) and footshock (10 to 20 s; square) in shock activated and shock inhibited groups. Data are means ± SEM. Shock activated (*n* = 4 to 5 mice), CT14, **P = 0.005 (F). CT22, nonsignificant (G). CT6, nonsignificant (H). Shock inhibited (*n* = 3 mice), CT14, nonsignificant (F). CT22, nonsignificant (G). CT6, *P = 0.004 (H). Two-tailed paired-sample *t* test. (**I** to **K**) Latency to reach peak activation (green) or inhibition (blue) from the onset of footshock. Data are means ± SEM. CT14, shock activated, (*n* = 5 mice), shock inhibited (*n* = 3 mice), ***P = 0.0009 (I). CT22, shock activated (*n* = 5 mice), shock inhibited (*n* = 3 mice), **P = 0.0036 (J). CT6, shock activated, (*n* = 4 mice), shock inhibited, (*n* = 3 mice), *P = 0.023 (K). Student’s *t* test. Light pulse (15 s) and footshock duration (2 s) are depicted by dotted line and shaded area. (**L** to **N**) Heatmaps of average calcium responses to light (five trials per mouse) and average GCaMP7s responses at CT14 (L), CT22 (M), and CT6 (N). Continuous yellow in heatmaps represents time points where response exceeded maximum *z*-score. ITI, intertrial interval.

Given that stressors influence the central pacemaker, we investigated whether the SCN acutely responds to footshocks. We injected an adeno-associated virus (AAV) encoding a Cre-dependent fluorescent calcium indicator, GCaMP7s, into the SCN of NMS-Cre mice. NMS neurons not only constitute 40% of all SCN neurons but are also further subdivided into arginine vasopressin (AVP)-expressing neurons in the SCN shell and vasoactive intestinal peptide (VIP)-expressing neurons in the SCN core, thereby representing the two major anatomical subdivisions of the SCN (*42*). Using fiber photometry, we recorded the SCN^NMS+^ population responses to footshocks and light under DD at CT14, CT22, and CT6 (Fig. 3, A and B). The same mice were tested at all CT times in a randomized order.

Aversive stimuli elicited net increases or decreases in the fluorescence signal recorded from SCN^NMS*+*^ neurons at all three CT times (Fig. 3, C to H; and fig. S2, B and C). Specifically, at both CT14 and CT22, footshocks produced net increases in GCaMP fluorescence (shock activated) in five of the eight mice, whereas three of the eight exhibited net decreases (shock inhibited; Fig. 3, C, F, D, and G; and fig. S2, B and C). At CT6, a weak net increase was observed in four of the seven mice, while three of the seven showed net decreases (Fig. 3, E and H; and fig. S2, B and C). Mice that displayed a particular response pattern (increase versus decrease) did so consistently across trials and CT times (fig. S2, B and C). Overall, SCN^NMS*+*^ photometric signals from shock-activated mice were higher at CT14 relative to CT6, with CT22 measures falling between these two time points but without reaching statistical significance (fig. S2D). In the shock-inhibited group, the decreases remained consistent across all CT times (fig. S2D). Notably, the latency to peak for the decreases in fluorescence was significantly longer than that of the net increases at each CT time tested (Fig. 3, I to K). These observations indicate that footshock stress broadly affects SCN^NMS*+*^ activity dynamics, warranting further examination with more specific neuronal markers.

In contrast to footshocks, light robustly increased SCN^NMS+^ fluorescence at CT14 and CT22, in line with a previous report (Fig. 3, L and M, and fig. S2E) (*49*). However, at CT6, light produced biphasic patterns, with brief early increases followed by delayed decreases (Fig. 3N and fig. S2E). To explore whether the subgroups that differed in their response to footshocks (shock activated versus shock inhibited) also diverged in their response to light, we analyzed light-evoked signals separately for each subgroup (fig. S2, F to K). At CT14 and CT22, mice classified as shock activated exhibited a greater light-induced increase in fluorescence compared to the shock-inhibited group (fig. S2, F to I). At CT6, SCN^NMS*+*^ neurons of shock-activated mice showed only small increases in responses to light stimulus, whereas the shock-inhibited mice displayed net decreases in fluorescence (fig. S2, J and K). Given that the SCN is predominantly gamma-aminobutyric acid (GABA)ergic (*50*–*52*), these delayed decreases to both footshock and light may reflect local feedforward inhibition. This heterogeneity does not appear to be attributable to differences in optic fiber placement or GCaMP7s expression (fig. S2C). We next examined how light modulates SCN^NMS+^ responses to footshocks. At CT14, the presence of light resulted in SCN^NMS+^ neurons exhibiting larger fluorescence increases in response to footshocks compared to controls in darkness (fig. S3, A to E and I). Notably, neurons that previously displayed decreases in calcium signal showed an increase under light conditions (fig. S3, F to I). Overall, these observations show that SCN neurons respond directly to both aversive and photic stimuli.

### SCN-projecting aPVT neurons are activated by aversive stimuli

**Fig. 4.**
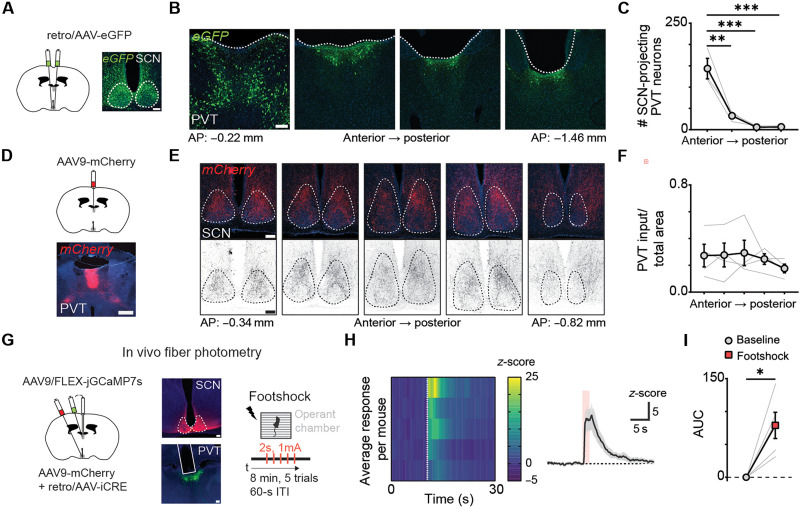
SCN-projecting PVT neurons are activated by footshocks. (**A**) Schematic of the retrograde tracing strategy used for labeling SCN-projecting PVT neurons (left) and representative images showing injection sites for eGFP in the SCN (right). Scale bar, 100 μm. (**B**) Representative images showing eGFP expression in PVT neurons across anteroposterior axis. Scale bar, 100 μm. (**C**) Quantification of PVT neurons projecting to SCN. Data are means ± SEM (*n* = 3 mice). **P = 0.0012; ***P = 0.0003, one-way ANOVA, Tukey’s test. (**D**) Schematic of the viral vector strategy for anterograde tracing of PVT projections and representative image showing injection site for *mCherry* in the PVT. Scale bar, 500 μm. (**E**) Representative images showing CAMKII-mCherry expression in axon terminals within the SCN along the anteroposterior axis. Scale bar, 100 μm. (**F**) Quantification of the density of PVT axonal inputs in the SCN. Data are means ± SEM (*n* = 4 mice). Not significant, one-way ANOVA, Tukey’s test. (**G**) Schematic of tracing strategy to specifically express GCaMP7s in PVT neurons that project to the SCN (left). Representative image of injection site in the SCN and GCaMP7s expression in SCN-projecting PVT neurons and fiber placement. Scale bar, 100 μm (middle). Schematic of the footshock session used in fiber photometry experiments (right) (**H**) Heatmap of average *z*-score of ∆*F*/*F* of five footshock trials per mouse and average GCaMP7s responses to footshocks (*n* = 5 mice). Footshock duration (2 s) is depicted by shaded area. (**I**) Area under the curve (AUC) comparison between baseline (0 to 10 s; black) and footshock (10 to 20s; gray) groups. Data are means ± SEM (*n* = 5 mice). *P = 0.0169, two-tailed paired-sample *t* test. ITI, intertrial interval.

To determine which brain regions convey aversive information to the central pacemaker, we injected a retrograde tracing virus expressing enhanced green fluorescent protein (eGFP) into the SCN of WT mice (Fig. 4A). Consistent with a previous study (*53*), we observed retrogradely labeled neurons largely confined to the aPVT (Fig. 4, B and C), ventral premammillary nucleus, zona incerta, dorsal raphe, and periaqueductal of gray (fig. S4A). The PVT has previously been recognized for its sensitivity to multimodal stressors, including footshocks, and its crucial function in regulating both neuroendocrine and behavioral adaptations to stress (*54*). Given this, we focused on further characterizing the anatomical projections from the aPVT to the SCN by injecting an anterograde tracing virus expressing mCherry into the aPVT of WT mice and quantified the distribution of efferent projections across the anterior-posterior extent of the SCN (Fig. 4D). We observed a relatively uniform distribution of PVT fibers throughout the anterior and posterior regions of the SCN (Fig. 4, E and F), confirming that the aPVT projects broadly to the SCN.

To determine whether the PVT transmit stress-related signals to the SCN, we specifically labeled SCN-projecting PVT neurons by injecting a retrogradely transported Cre-carrying vector into the SCN and a Cre-dependent GCaMP7s virus into the aPVT and implanted an optical fiber above the aPVT (Fig. 4G). Fiber photometry–mediated bulk calcium imaging demonstrated that SCN-projecting aPVT neurons exhibited robust increases in fluorescence in response to footshocks (Fig. 4, H and I), thus supporting the notion that the aPVT might be a conduit for transmitting aversive signals to the central pacemaker.

Recent studies demonstrated that the PVT comprises molecularly distinct neurons (*55*, *56*) that display divergent responses to aversive stimuli, including footshocks (*57*). In particular, anterior-biased neuronal populations of the PVT can be divided into anterolateral and anteromedial subtypes based on their expression of the genes *Npffr1* and *Hcrtr1*, respectively (*55*). Thus, to further define the aPVT-SCN connection, we retrogradely labeled SCN-projecting PVT with eGFP and performed RNA fluorescence in situ hybridization against *Hcrtr1* and *Npffr1* (fig. S4B). Of the total *eGFP^+^* cells, most cells expressed *Npffr1* (60%), a small fraction of which co-expressed *Hcrtr1^+^* (16% of all eGFP^+^ cells) (fig. S4, C and D). A small population of eGFP^+^ cells solely expressed *Hcrtr1* (16%; fig. S4, C and D). Approximately 24% of SCN-projecting PVT cells did not express either mRNA (fig. S4, C and D). These data show that molecularly heterogeneous subpopulations of aPVT neurons project to the SCN and, unlike cortically projecting aPVT neurons (*57*), are activated by footshocks.

### PVT is required for SCN’s response to aversive stimuli

**Fig. 5.**
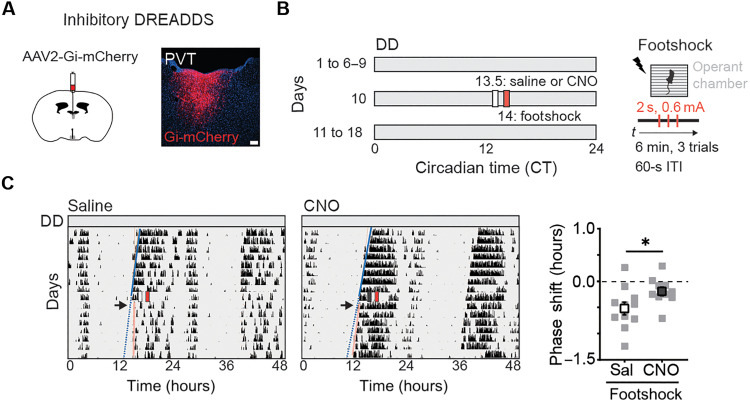
PVT is necessary for the effects of aversive stimuli on the central pacemaker. (**A**) Schematic of the stereotaxic injection to target expression of inhibitory DREADDS to aPVT neurons (left). Representative image of hM4D(Gi)-mCherry expression in the aPVT (right). Scale bar, 100 μm. (**B**) Schematic of experimental paradigm; mice were placed in constant darkness (DD) for 6 to 9 days, and, then, at CT13.5, saline or CNO was injected intraperitoneally. Thirty minutes later, mice were subjected to a footshock session and allowed to free run for an additional 7 days. (**C**) Running-wheel activity profiles of control mice injected with saline (white rectangle) or CNO (dark gray rectangle) and subsequently subjected to a footshock session (red rectangle) at CT14 (left). Quantification of the phase shifts (hours) in response to footshocks in saline (Sal)– and CNO-injected mice at CT14 (right). Data are means ± SEM (*n* = 10 to 11 mice). *P = 0.04, Student’s *t* test. ITI, intertrial interval.

Our results show that both SCN-projecting aPVT neurons and SCN^NMS*+*^ neurons respond to aversive stimuli, suggesting a central role for the aPVT in mediating the interaction between stressors and the central pacemaker. To evaluate whether the aPVT is necessary for the SCN-dependent phase delays induced by footshocks, WT mice were injected with an a Cre-dependent AAV encoding inhibitory (Gi) DREADDs (designer receptors exclusively activated by designer drugs) (*58*), enabling chemogenetic silencing of aPVT neurons upon administration of the DREADD ligand clozapine-*N*-oxide (CNO) (Fig. 5A). Two weeks after injection, mice were placed in constant dark conditions for 7 days and then received an intraperitoneal injection of CNO (or saline control) at CT13.5 to selectively inhibit aPVT neurons, 30 min before the footshock session (CT14) (Fig. 5B). Locomotor activity rhythms were monitored for 7 days posttreatment. Silencing PVT neurons resulted in a reduction in the phase delays caused by footshocks compared to saline-injected controls (Fig. 5C). Together, these results demonstrate that the aPVT is necessary for mediating the phase-shifting effects of stressors on the central pacemaker.

### PVT neurons send glutamatergic inputs to the SCN to drive inhibitory microcircuits

**Fig. 6.**
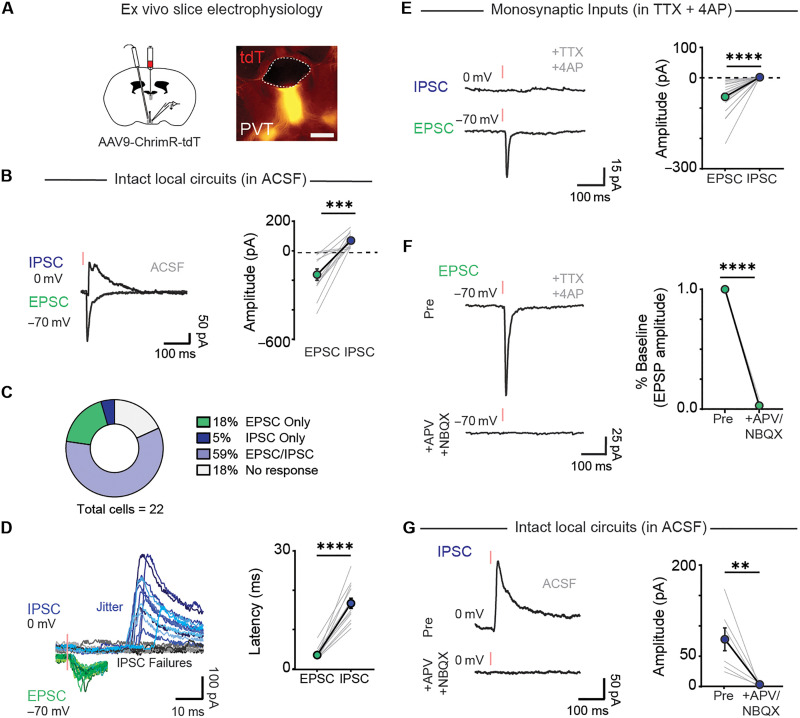
PVT neurons send glutamatergic inputs to SCN to recruit local inhibitory microcircuits. (**A**) Schematic of anterograde tracing strategy used for expressing ChrimR-TdT in aPVT axons innervating the SCN and image depicting TdT expression in the PVT. Scale bar, 200 μm. (**B**) Sample trace showing optically evoked responses in the same SCN neurons following aPVT optogenetic stimulation: IPSCs (blue) and EPSC (green). Comparison between EPSC and IPSC amplitude from cells that display both EPSC/IPSC (right). Data are means ± SEM (*n* = 13 neurons and *n* = 7 mice). ***P = 0.0001, two-tailed paired *t* test. (**C**) Proportion of SCN neurons that receive excitatory/or inhibitory inputs from aPVT (*n* = 22 neurons). (**D**) Sample trace of optically evoked EPSC (green) and delayed IPSC (blue) elicited in SCN neurons (left). IPSC failures are represented by black and gray traces (right). Synaptic latency of EPSC (*n* = 17) and IPSC (*n* = 14) after optogenetic stimulation of aPVT axons (left). Data are means ± SEM. ****P < 0.0001, two-tailed paired *t* test. (**E**) Sample trace showing optically evoked responses in SCN neurons in the presence of TTX and 4-AP (right). Comparison between EPSC and IPSC amplitude (right). Data are means ± SEM (*n* = 21 neurons and *n* = 5 mice). ****P < 0.0001, two-tailed paired *t* test. (**F**) Sample trace showing optically evoked EPSC in SCN neurons pre and post bath application of NBQX and D-AP5 (left). Amplitude in SCN neurons receiving excitatory input from PVT pre– and post–D-AP5 and NBQX application (right). Data are means ± SEM (*n* = 5 neurons, *n* = 5 slices, and *n* = 5 mice). ****P < 0.0001, two-tailed paired *t* test. (**G**) Sample trace of SCN neurons showing optically evoked IPSC (left). IPSC amplitude pre and post application of NBQX and D-APV (right). Data are means ± SEM (*n* = 7 neurons and *n* = 6 mice). **P = 0.0072, two-tailed paired *t* test.

While SCN neurons displayed divergent calcium responses to footshocks, specifically net increases in some mice and delayed decreases in others, SCN-projecting aPVT neurons are selectively activated by this stimulus. A prior study using electrical stimulation reported that the PVT releases both glutamate and GABA to the SCN (*39*), which may support the response profiles observed in the SCN. To determine whether aPVT neurons directly innervate SCN^NMS*+*^ neurons, we performed monosynaptic rabies tracing and found that neurons within the anterolateral PVT are readily labeled by this manipulation (fig. S5). To further characterize the PVT-SCN circuit while overcoming the limitations of prior studies using electrical stimulation, we used channelrhodopsin 2 (ChR2)–assisted circuit mapping combined with whole-cell patch-clamp electrophysiology. We delivered AAVs carrying the red-shifted ChR2 variant ChrimsonR (*59*) (AAV9-hsyn-ChrimR-TdT) to the aPVT of WT mice (Fig. 6A). We then prepared brain slices and recorded optogenetic-evoked synaptic responses onto SCN neurons. Optogenetic stimulation of aPVT axons readily evoked postsynaptic currents in SCN neurons (82% of all cells recorded; Fig. 6, B and C). Most cells exhibited both excitatory postsynaptic currents (EPSCs) and inhibitory postsynaptic currents (IPSCs; 59%), while a smaller proportion of cells showed only EPSCs (18%) or only IPSCs (5%) (Fig. 6, B and C). The onset latency for IPSCs (~16 ± SEM ms) was significantly longer than that of EPSCs (~3 ± SEM ms) for all cells (Fig. 6D). Considering that the SCN is predominantly a GABAergic nucleus (*50*–*52*), our data suggest that the EPSCs arise from monosynaptic aPVT inputs that recruit local inhibition within the SCN.

When we isolated monosynaptic inputs from the PVT using tetrodotoxin (TTX) and 4-aminopyridine (4-AP) in the extracellular solution (Fig. 6E), we observed that optogenetic stimulation of aPVT axons readily evoked EPSCs in SCN neurons, but no inhibitory currents were observed (Fig. 5E). EPSCs were completely blocked by bath application of 2,3-dihydroxy-6-nitro-7-sulfamoylbenzo[*f*]quinoxaline (NBQX) and d-(-)-2-amino-5-phosphonopentanoic acid (D-AP5), which block AMPA and *N*-methyl-d-aspartate (NMDA) receptors, respectively (Fig. 6F), showing that, unlike previously reported, the aPVT sends only excitatory glutamatergic inputs to the SCN. Next, to investigate whether aPVT drives local inhibition in the SCN, we isolated IPSC with an intact local circuit and bath applied of NBQX and D-AP5 (Fig. 6G). Blocking glutamatergic inputs abolished the evoked IPSC in SCN neurons. Collectively, our results demonstrate that inhibitory currents evoked by PVT stimulation are a result of polysynaptic inhibition arising within the SCN.

### Inhibitory local SCN network modulates SCN response to stressors

The SCN is a highly interconnected nucleus, with its network providing robustness in circadian rhythmicity and buffering against certain non-photic cues, such as temperature changes (*60*). Given this and our finding that the aPVT recruits a feedforward inhibitory microcircuit, we sought to investigate how SCN network dynamics influence its neuronal response to stressors.

We co-injected the SCN of NMS-Cre mice with a Cre-dependent GCaMP7s virus and a Cre-dependent AAV encoding inhibitory (Gi) DREADDs, enabling chemogenetic silencing of SCN^NMS*+*^ neurons (fig. S6A). Using fiber photometry, we recorded SCN^NMS+^ population responses to footshocks at CT14, following an intraperitoneal injection of either saline or CNO 30 min before the session (fig. S6B). Silencing a proportion of SCN^NMS*+*^ neurons enhanced calcium responses to footshock compared to saline controls (fig. S6, C and D). These findings suggest that the intrinsic GABAergic network within the SCN (*50*–*52*) exerts inhibitory control over shock-responsive neurons, thereby dampening SCN responses to stressors under normal conditions.

**Fig. 7.**
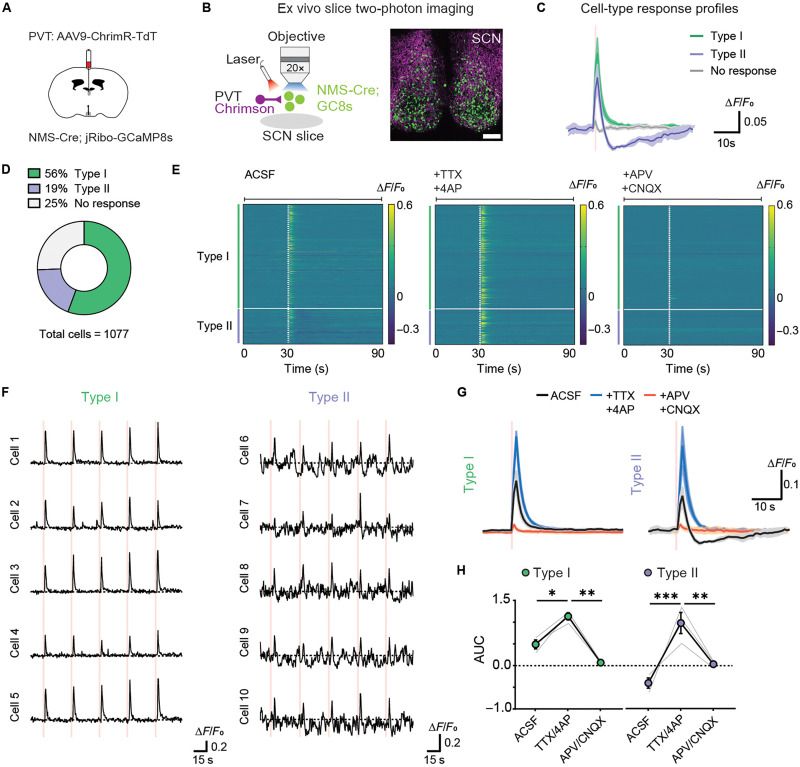
Local inhibitory microcircuit shapes SCN response to PVT inputs. (**A**) Schematic of the viral vector strategies used for combined two-photon Ca^2+^ imaging of GCaMP8s fluorescence from SCN^NMS*+*^ neurons and optogenetic manipulation of PVT terminals in the SCN. (**B**) Schematic (left) and representative image of SCN brain slice with expression of ChrimR-TdT in PVT axons (magenta) and GCaMP8s (green) in SCN^NMS*+*^ neurons (right). Scale bar, 100 μm. (**C**) Average fluorescence responses of SCN^NMS*+*^ neurons, categorized by response type, to aPVT optogenetic stimulation at CT14. Type I represents SCN^NMS+^ neurons that showed net increases, and type II represents SCN^NMS+^ neurons that showed brief increases followed by decreases in response to aPVT optogenetic stimulation. (**D**) Proportion of SCN neurons classified as type I, type II, or nonresponsive (*n* = 1077 neurons, *n* = 3 slices, and *n* = 2 mice). (**E**) Heatmap showing average GCaMP8s fluorescence (∆*F*/*F*, five trials) per cell in response to optogenetic stimulation (200 ms, 550 nm at 20 Hz; dotted line) in the presence of ACSF (left), TTX (500 nM), and 4-AP (100 μM, left), and D-AP5 (50 μM) and CBQX (10 μM, right). (**F**) Example traces showing individual trials per cell for the two distinct response types in SCN^NMS*+*^ neurons following optogenetic stimulation. (**G**) Average GCaMP8s fluorescence responses to optogenetic stimulation of PVT terminals in ACSF (black) or in the presence of TTX/4-AP (blue), or APV/CNQX (orange). Optogenetic stimulation (200 ms) is depicted by shaded area. (**H**) Quantification of area under the curve (AUC, 30 to 90 s, bottom) for each response type in ACSF, TTX/4-AP, and APV/CNQX. Data are means ± SEM. Type I, *P = 0.033, **P = 0.002; type II, **P = 0.004, ***P = 0.0003; two-way ANOVA, Šidák’s test.

To further dissect the role of SCN network dynamics in modulating stress inputs from the aPVT, we delivered a virus driving constitutive expression of ChrimsonR in the aPVT of NMS-Cre;GCaMP8s mice (Fig. 7A). After a 3-week recovery period to allow for viral expression, mice were placed in DD for 24 hours to avoid any light mediated neuronal effects. Brain slices containing the aPVT and SCN were then prepared at CT14 for ex vivo two-photon imaging of SCN^NMS*+*^ neurons, combined with optogenetic stimulation of PVT-SCN axon terminals (Fig. 7B).

Brief 200-ms trains of optical stimulation of PVT-SCN terminals (20 Hz; 1-ms pulse) elicited two types of responses in SCN^NMS*+*^ neurons: type I, characterized by net increases, and type II, marked by brief increases followed by decreases (Fig. 7, C to G). While neurons of both response types were distributed throughout the SCN, type I neurons appeared more densely localized in the shell, whereas type II appeared more concentrated in the core (fig. S6E). Consistent with our electrophysiological experiments, bath application of TTX and 4-AP to isolate monosynaptic inputs abolished the decreases observed in the type II responses (Fig. 7, E, G, and H). Moreover, consistent with the potentiation of shock responses observed in vivo, blocking SCN network activity with TTX and 4-AP results in stronger activation of SCN^NMS*+*^ neurons in both response types (Fig. 7, E, G, and H). As expected, all PVT-evoked changes in fluorescence were eliminated by application of AMPA and NMDA receptor antagonists (Fig. 7, E, G, and H). These data, combined with our electrophysiology recordings, demonstrate that stimulation of PVT-SCN terminals elicits initial excitatory responses followed by feedforward inhibitory responses in a subset of SCN^NMS+^ neurons and highlights the role of the local SCN network in shaping the SCN’s response to stressors.

### Stress and light are differentially encoded in AVP- and VIP-expressing SCN neurons

**Fig. 8.**
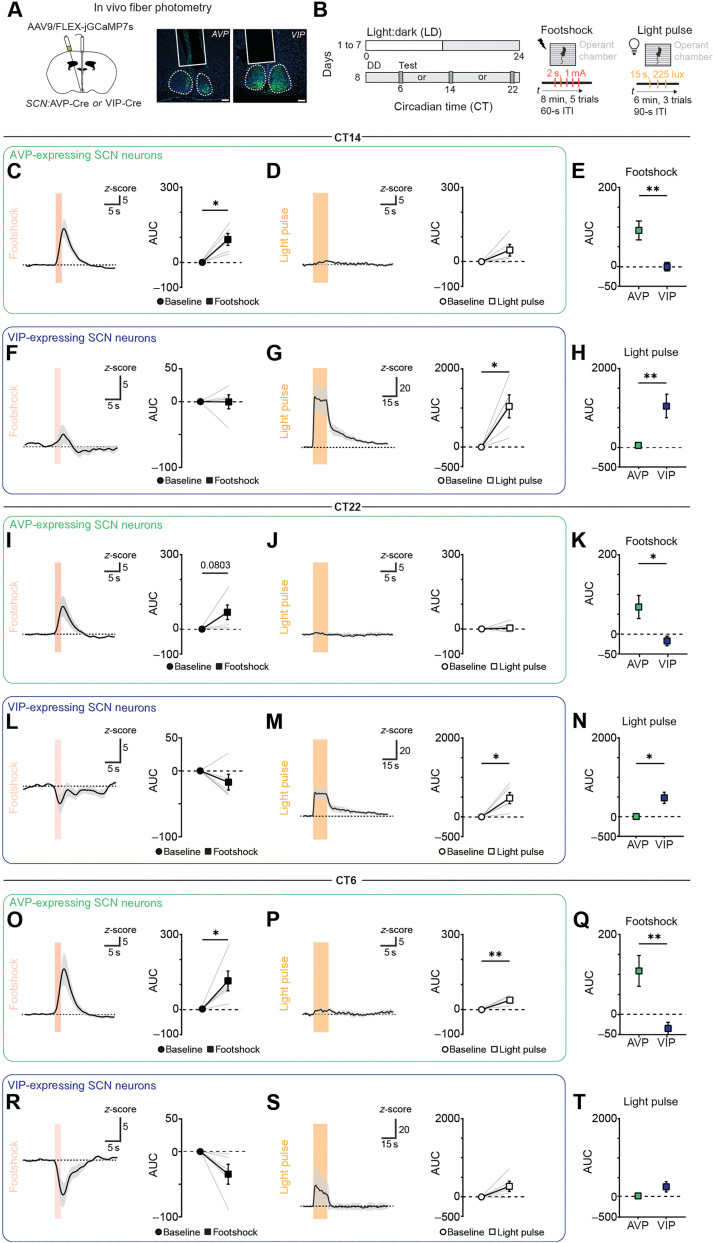
The SCN differentially encodes stress and light information in SCN^AVP+^ and SCN^VIP+^ neurons. (**A**) Schematic of stereotaxic injection strategy to selectively express GCaMP7s in SCN^AVP*+*^ and SCN^VIP*+*^ neurons, with representative images showing GCaMP7s expression and fiber placement. Scale bars, 100 μm. (**B**) Experimental paradigm. (**C** and **D**) Average calcium responses in SCN^AVP*+*^ neurons to footshocks (C) and light (D) at CT14. *P = 0.019. (**E**) Comparison of footshock responses between SCN^AVP*+*^ and SCN^VIP*+*^ neurons at CT14. **P = 0.008. (**F** and **G**) Average calcium responses in SCN^VIP*+*^ to footshocks (F) and light (G) at CT14. *P = 0.023. (**H**) Comparison of light responses between SCN^AVP*+*^ and SCN^VIP*+*^ neurons at CT14. **P = 0.009. (**I** and **J**) Average calcium responses in SCN^AVP*+*^ to footshocks (I) and light (J) at CT22. (**K**) Comparison of footshock responses between SCN^AVP*+*^ and SCN^VIP*+*^ neurons at CT22. *P = 0.027. (**L** and **M**) Average calcium responses in SCN^VIP*+*^ to footshocks (L) and light (M) at CT22. *P = 0.028. (**N**) Comparison of light responses between SCN^AVP*+*^ and SCN^VIP*+*^ neurons at CT22. *P = 0.010. (**O** and **P**) Average calcium responses in SCN^AVP*+*^ to footshocks (O) and light (P) at CT6. *P = 0.046; **P = 0.008. (**Q**) Comparison of footshock responses between SCN^AVP*+*^ and SCN^VIP*+*^ neurons at CT6. **P = 0.008. (**R** and **S**) Average calcium responses in SCN^VIP*+*^ to footshocks (R) and light (S) at CT6. (**T**) Comparison of light responses between SCN^AVP*+*^ and SCN^VIP*+*^ neurons at CT6. Line graphs depict GCaMP7s response quantification using the area under the curve (AUC) during baseline (0 to 10 s), footshock (10 to 20 s), and light (10 to 40 s). Footshock duration (2 s) is indicated by a red-shaded area, and light pulse (15 s) is indicated by an orange-shaded area. Baseline-versus-stimulus responses were analyzed using a two-tailed paired-sample *t* test. Comparisons between AVP^+^ and VIP^+^ neurons were performed using unpaired Student’s *t* test. ITI, intertrial interval.

Our results suggest that the complex responses of SCN^NMS*+*^ neurons to stress or aPVT input likely reflect an underlying heterogeneity, as most of SCN^NMS*+*^ neurons can be divided into AVP-expressing cells (in the SCN shell) and VIP-expressing cells (in the SCN core) (*42*). This spatial distinction aligns with our ex vivo findings and the denser aPVT innervation of the SCN shell. Therefore, we used fiber photometry to monitor GCaMP7s fluorescence in SCN^AVP*+*^ and SCN^VIP*+*^ neurons at CT14, CT22, and CT6 following footshock or light stimulation, enabling a direct comparison of the two populations (Fig. 8, A and B).

In vivo calcium imaging showed that SCN^AVP*+*^ neurons exhibited robust increases in calcium signal at all three circadian times, with minimal evidence of fluorescence decreases (Fig. 8, C, I, and O; and fig. S7, A, E, and I). In contrast, SCN^VIP*+*^ neurons showed smaller increases and pronounced decreases in response to footshock (Fig. 8, F, L, and R; and fig. S7, C, G, and K). Specifically, SCN^VIP*+*^ neurons displayed only a modest increase at CT14 (Fig. 8, F and E, and fig. S7C) but exhibited clear decreases at CT22 and CT6 (Fig. 8, K to L and Q to R; and fig. S7, G and K). These findings indicate that distinct SCN subpopulations underlie the divergent response profiles to stressors.

In contrast, previous research demonstrated that VIP-expressing neurons, which receive dense retinal innervation, respond robustly to light stimuli, whereas AVP-expressing neurons with minimal retinal input may not (*37*, *61*–*63*). Consistent with that, our recordings revealed minimal light-evoked activity in SCN^AVP*+*^ neurons but large increases in fluorescence in SCN^VIP*+*^ neurons (Fig. 8, D, G to H, J, M to N, P, and S to T; and fig. S7, B, D, J, L, F, and H). Together, these data establish that stressors preferentially recruit the shell SCN^AVP*+*^ population, whereas light predominantly activates the core SCN^VIP*+*^ population. This functional segregation highlights how the SCN leverages distinct subpopulations to encode multiple environmental cues, providing a mechanistic basis for circadian adaptations to both stress and light stimuli.

## DISCUSSION

In this study, we show that aversive stimuli can reprogram the central pacemaker via an aPVT-to-SCN (aPVT-SCN) circuit. The aPVT sends excitatory glutamatergic signals to the SCN, similar to retinal projections. We propose that stressors and light are encoded by overlapping but distinct SCN microcircuits, as evidenced by differences in the phase response strength and direction, and the additive or subtractive phase changes when both inputs are combined. The results offer insights into the behavior and circuit dynamics by which photic and non-photic signals are integrated across the daily cycle.

### Stressors reprogram the central pacemaker

Some studies have examined how non-photic cues affect the central circadian pacemaker (*13*, *25*). However, only a few have identified specific cues that adjust the SCN and the neural circuits that mediate this response (*47*, *48*, *64*–*66*). Arousal and exercise have been shown to phase advance circadian rhythms during the inactive period, with evidence suggesting roles for the intergeniculate leaflet and dorsal raphe in mediating these responses, respectively (*22*–*24*, *48*, *66*). Other studies have manipulated brain regions known to project to the SCN and characterized their effects on light entrainment but have not identified the specific stimuli that activate the circuits impinging on the central pacemaker (*43*, *46*). Studies exploring contributions of specific non-photic stimuli, such as temperature, have found that the SCN is resistant to temperature changes and only responds to this stimulus when intracellular communication within the SCN is disrupted (*60*).

Our findings demonstrate that aversive stimuli can induce phase shifts when the central pacemaker is intact, as evidenced by changes in the phase of locomotor activity and SCN calcium rhythms. Specifically, we find that footshocks and forced-swim stress caused phase delays in locomotor activity rhythms at CT14, similar to the effects of light but with a smaller magnitude. At CT22, stressors caused phase delays, whereas light induced phase advances. At CT6, stressors did not affect the phase of the central pacemaker, similar to light. These results indicate that the effects of non-photic cues, thought to phase advance the central pacemaker during the inactive period, cannot be generalized to all stimuli (*22*–*24*). Furthermore, pairing light with footshocks produced distinct effects on the magnitude and direction of phase shifts compared to light alone. Collectively, our data establish that stressors function as true zeitgebers to the circadian system.

A common phenomenon in stress research is that animals can show resilience or vulnerability to stress depending on genetic and/or environmental factors (*67*). From this perspective, variability in phase delays in response to footshock among individual animals may reflect inherent differences in susceptibility scores across our experimental population.

### Aversive stimuli engage inhibitory SCN microcircuits

Although, at the population level, infralimbic-projecting aPVT neurons are inhibited by footshocks (*57*), here, we find that SCN-projecting aPVT neurons are activated by aversive stimuli and send only glutamatergic inputs to the central pacemaker, similar to those from the retina (*68*–*70*). Neuronal activity recordings from SCN^NMS*+*^ neurons in response to footshocks resulted in net increases in some mice and decreases in others at all CT times. We interpret the inhibitory component of the footshock response as an intrinsic feature of the SCN circuitry, given that the decreases in fluorescence in response to footshocks exhibited longer latencies compared to the increases. This notion is further supported by our electrophysiology and ex vivo Ca^2+^ imaging experiments demonstrating that PVT optogenetic stimulation can elicit monosynaptic excitation and feedforward inhibition in SCN neurons. Furthermore, blocking monosynaptic inputs from the PVT abolished inhibitory responses and uncovered further excitation in SCN^NMS*+*^ neurons. These results highlight the polysynaptic nature of PVT-driven inhibition in the SCN and the contributions of the SCN local inhibitory circuit in shaping SCN responses to footshocks.

The distinct responses in shock-activated and shock-inhibited groups stem from footshock information being encoded within subpopulations of SCN^NMS*+*^ neurons. Although our in vivo studies did not reveal differences in GCaMP expression or fiber placement that could indicate spatial differences between activation and inhibition, our ex vivo slice two-photon experiments as well as fiber photometry experiments recording from SCN^AVP*+*^ and SCN^VIP*+*^ neurons show that distinct population of SCN neurons are differentially responsive to footshocks. We, therefore, propose a model in which stressors activate SCN-projecting PVT neurons. In turn, these neurons send excitatory glutamatergic inputs specifically to the SCN shell, where SCN^NMS*+*^ neurons express AVP. These shell neurons may cross-inhibit other SCN neurons in the shell and also inhibit SCN core neurons, such as SCN^NMS*+*^ neurons that express VIP (fig. S8A).

Previous studies have shown that spontaneous SCN activity is higher during mouse’s active period (night, CT0 to CT12) and lower during its inactive period (day, CT12 to CT24) (*63*, *71*). In line with these findings, our data show that calcium levels in SCN^NMS*+*^ neurons peak at CT6, reach their lowest at CT14, and are intermediate at CT22 (fig. S1F). We propose that the daily changes in SCN spontaneous activity shapes the excitatory/inhibitory (E/I) balance of the SCN’s response to incoming stimuli, thereby influencing the magnitude of phase shifts. During the night, when SCN spontaneous activity is low, a shift toward a higher excitatory imbalance causes larger phase delays. Conversely, during the day, when SCN activity is high, a more balanced E/I ratio prevents significant phase shifts (fig. S8B). Our data support this model by showing that the net response of SCN neurons to both footshocks and light, as represented by SCN^NMS+^ neuronal activity, is predominantly excitatory at CT14 and CT22 and shifts to weaker excitation or becomes inhibitory at CT6. The net E/I balance is achieved through the differential engagement of SCN neuronal subtypes in response to distinct stimuli. Correspondingly, our results show that SCN^VIP+^ neurons respond more strongly to light at CT14 compared to that at CT6, while SCN^AVP+^ neurons show minimal responses to light at all circadian times. In contrast, SCN^AVP+^ neurons show consistent increases in response to footshocks at all circadian times, whereas SCN^VIP+^ neurons exhibit large decreases at CT6. Together, the intrinsic rhythms of SCN neurons and the selective engagement of distinct circuits by different stimuli shape the net E/I balance, which is observed in the response of SCN^NMS+^ neurons to light and footshocks, determining the magnitude of phase shifts. Furthermore, our findings suggest that SCN^VIP+^ neurons may play a critical role in gating the response to incoming stimuli, even when they are not the primary neurons activated by the environmental cue.

In agreement with our model, our data show that pairing light with footshocks at CT14 abolishes the decreases in fluorescence observed in the recorded SCN^NMS*+*^ neurons in response to footshocks in darkness, suggesting that light input directly converges onto the SCN microcircuit recruited by stressors. This convergence enhances activation and suppresses inhibition, resulting in a stronger phase delay when both stimuli are paired (fig. S8B). These findings illustrate that, by dynamically shifting the E/I balance, the SCN integrates environmental cues through specific microcircuits to fine-tune the strength of the phase shift.

### PVT-SCN axis: A hub for the interaction between homeostatic and circadian systems

Recent research has identified the PVT as a critical node for integrating homeostatic challenges such as hunger, threats, and temperature through inputs emerging from the brainstem and hypothalamus (*26*). In turn, the PVT guides the selection of goal-oriented actions that promote homeostasis (*26*). Our study demonstrates that neurons in the aPVT relay acute stress-related information to the SCN, causing shifts in the phase of the central pacemaker. Silencing aPVT neurons reduces the phase delays in response to footshocks. These results implicate the aPVT in mediating the effects of stressors on the central pacemaker. A potential limitation of the current study is that broad silencing of aPVT neurons may not be specific to aPVT-SCN pathway. However, we believe that our approach primarily targets aPVT-SCN neurons, as these neurons are selectively activated by shocks, whereas most of cortical-projecting neurons in this region are inhibited by this stimulus (*57*). Moreover, these SCN-projecting aPVT neurons relay stress-related signals via glutamatergic inputs to the SCN.

We propose that amid environmental challenges or stressors, the aPVT may serve to orchestrate shifts in circadian behavioral patterns to avoid threats through its downstream projections to the SCN. Several studies have shown that the SCN also projects back to the PVT, likely conveying time-of-day information (*37*, *72*, *73*). PVT neuronal activity is antiphase with the SCN (*74*, *75*), implicating GABAergic inhibition. This bidirectional communication may allow the PVT not only to broadcast stress-related signals to the hypothalamus but also to receive circadian information to drive appropriate goal-directed responses though the forebrain and limbic systems (*26*). Within this context, we propose that the aPVT-SCN connection serves as a channel through which the homeostatic and circadian systems interact, forming a hub essential for survival.

Our work uncovers an anterior PVT-SCN circuit essential for the effects of stressors on the central pacemaker and reveals that photic and non-photic cues are integrated within the SCN. These discoveries lay the foundation for investigating the neuronal basis of psychiatric disorders, which are strongly associated with chronic stress exposure and circadian disruption.

## MATERIALS AND METHODS

### Animals

All procedures were performed in accordance with the Guide for the Care and Use of Laboratory Animals and were approved by the National Institute of Mental Health (NIMH) Animal Care and Use Committee. Mice were housed under a 12-hour light (L):12-hour dark (D) cycle with food and water ad libitum. The following mouse lines from the Jackson Laboratory were used: C57BL/6J (strain no. 000664), NMS-Cre (strain no. 027205), TIGRE2-RiboL1-jGCaMP8s-IRES-tTA2 (strain no. 039267), AVP-Cre (strain no. 023530), and VIP-Cre (strain no. 031628). Male mice 12 to 26 week of age were used for all experiments. Animals were randomly allocated to the different experimental conditions reported in this study.

### Viral vectors

AAVrg-hSyn-eGFP (plasmid no. 50465), AAV9-hSyn-eGFP (plasmid no. 50465), AAV9-hSyn-mCherry and AAV8-hSyn-mCherry (plasmid no. 114472), AAV2 (retro)-CAG-iCre (plasmid no. 81070), AAV9-Syn-Flex-jGCaMP7s-WPRE (plasmid no. 104491), AAV2-hSyn-hM4D(Gi)-mCherry (plasmid no. 50475), and AAV8-hSyn-DIO-hM4D(Gi) (plasmid no. 44362) were produced by Addgene. AAV2-CaMKIIa-mCherry and AAV9-hSyn-ChrimsonR-tdTomato were produced by the Vector Core of the University of North Carolina (NC, USA). AAV8-hSyn-FLEX-TVA-P2A-eGFP-2A-oG was produced by Addgene (plasmid no. 85225). EnvA-SAD-ΔG-eGFP was produced by the Viral Vector Core of the Salk Institute for Biological Studies (CA, USA). All viral vectors were stored in aliquots at −80°C until use.

### Virus injection and stereotaxic surgery

Mice were deeply anesthetized, and AAVs or gDRabies were stereotaxically delivered using an AngleTwo stereotaxic device (Leica Biosystems). A 3.5-inch (8.89-cm) microcapillary pipette was pulled and loaded with the solution. Injections were performed using a microinjector (Nanojector II, Drummond Scientific Company) at the following stereotaxic coordinates: aPVT, −0.30 mm from bregma, 0.00 mm lateral from midline, and −3.80 and −3.60 mm vertical from cortical surface; SCN, +0.25 mm from bregma, +0.20 and −0.20 mm lateral from midline, and −5.74 mm vertical from cortical surface. For fiber photometry imaging, an optical fiber [400 μm outer diameter, 0.5 numerical aperture (NA); Doric Lenses] was implanted over the PVT or the SCN immediately after viral injections and cemented using Metabond Cement System (Parkell) and Jet Brand dental acrylic (Lang Dental Manufacturing). A heating pad was used to maintain the body temperature during surgeries. Animals received subcutaneous injections with Metacam (meloxicam, 1 to 2 mg kg^−1^) for analgesia and anti-inflammatory purposes. For anatomical analysis, mice were perfused at different times postinjection (2 to 3 weeks after AAV injection, or 5 days after gDRabies injection), and the brains were subsequently sectioned on a cryostat. Mice without correct targeting of optical fibers, tracers, or vectors were excluded from this study.

### Histology

Animals were deeply anesthetized with isoflurane (Dechra) and transcardially perfused with phosphate-buffered saline (PBS; 1×; pH 7.4), followed by paraformaldehyde (PFA; 4% in PBS). After extraction, brains were postfixed in 4% PFA at 4°C for 24 hours, and subsequently cryoprotected by transferring to a 30% PBS-buffered sucrose solution until brains were saturated (~48 hours). Coronal brain sections (40 to 50 μm) were cut using a cryostat (Leica Biosystems). For DREADD experiments, the signal was enhanced using immunohistochemistry. Sections were subsequently mounted onto glass slides and a coverslip was added using Fluoromount-G with 4′,6-diamidino-2-phenylindole (DAPI; Thermo Fisher Scientific). Images were acquired using an ECLIPSE Ti2 inverted microscope (Nikon). Image analysis and cell counting were performed using ImageJ software (Fiji, v1.54f).

### Immunofluorescence

Brain sections for DREADDs and rabies tracing experiments were washed and blocked for 1 hour in 10% bovine serum albumin [BSA; in 0.5% Phospate-buffered saline solution with Triton-X (PBST)] and then incubated using the following primary antibodies: 1:1000 goat tdTomato (LS-C340696-600, LSBio) and 1:1000 chicken eGFP (ab13970, Abcam), diluted in 2.5% BSA for 1 day at 4°C. Section were washed in 0.5% PBST and incubated in the following second antibodies for 1 hour: 1:500 donkey anti-goat Alexa Fluor 555 and 1:1000 goat anti-chicken Alexa Fluor 488 (Thermo Fisher Scientific). After staining with the secondary antibody, sections were then washed in 0.5% PBST and mounted on a slide using Fluoromount-G with DAPI (Invitrogen).

### Whole-cell patch-clamp slice electrophysiology

For all electrophysiological experiments, slices were prepared as previously described (*76*). Briefly, mice were anesthetized with isoflurane and decapitated, and their brains quickly were removed and placed in ice-cold *N*-methyl-d-glucamine (NMDG) cutting solution (92 mM NMDG, 2.5 mM KCl, 1.25 mM NaH_2_PO_4_, 10 mM MgSO_4_, 0.5 mM CaCl_2_, 30 mM NaHCO_3_, 20 mM glucose, 20 mM Hepes, 2 mM thiourea, 5 mM Na-ascorbate, and 3 mM Na-pyruvate, at pH 7.3 to 7.4, gassed with 95% O_2_ and 5% CO_2_). Coronal sections (250 μm thick) containing the SCN were cut in the ice-cold NMDG cutting solution using a VT1200S automated vibrating-blade microtome (Leica Biosystems) and were subsequently transferred to a heated incubation chamber containing the NMDG cutting solution at 34° to 35°C. After ~12 min, slices were transferred to a room temperature (20° to 24°C) holding chamber containing a Hepes-modified artificial cerebrospinal fluid (92 nM NaCl, 2.5 mM KCl, 1.25 mM NaH_2_PO_4_, 2 mM MgSO_4_, 2 mM CaCl_2_, 30 mM NaHCO_3_, 25 mM glucose, 20 mM Hepes, 2 mM thiourea, 5 mM Na-ascorbate, and 3 mM Na-pyruvate, at pH 7.3, gassed with 95% O_2_ and 5% CO_2_) and remained in the holding chamber until needed. For recordings, slices were transferred to the recording chamber and constantly supplied with a room-temperature artificial cerebrospinal fluid (ACSF; 118 mM NaCl, 2.5 mM KCl, 26.2 mM NaHCO_3_, 1 mM NaH_2_PO_4_, 20 mM glucose, 2 mM MgCl_2_, and 2 mM CaCl_2_, at pH 7.4, gassed with 95% O_2_ and 5% CO_2_).

Recordings from SCN neurons were obtained with a Multiclamp 700B amplifier (Molecular Devices). Recordings were done under visual guidance using an Olympus BX51 microscope with transmitted light illumination. Recordings were made in ACSF and pharmacological antagonists were added to the ACSF and bath applied. All recordings were made with borosilicate glass pipettes with tip resistance of 3 to 6 megohms. Access resistance was monitored throughout all recordings and recordings where access resistance increased above 30 megohms were not included in analyses.

To investigate synaptic connectivity from the aPVT to SCN, the red-shifted ChR2 variant ChrimsonR was expressed in aPVT via stereotaxic injection of AAV9-hSyn-ChrimsonR-tdTomato and allowed to express for 3 weeks. Optogenetically evoked synaptic responses were achieved by shining a green light-emitting diode (LED; 555 nm, pE-300^white^, CoolLED) over acute slices to drive ChrimsonR-expressing terminals. All recordings were done in voltage-clamp configuration using a Cs-based internal solution containing 117 mM Cs methanesulfonate, 10 mM Hepes, 2.5 mM MgCl_2_, 2 mM Na_2_–adenosine 5′-triphosphate, 0.4 mM Na_2_–guanosine 5′-triphosphate, 10 mM Na_2_-phosphocreatine, 0.6 mM EGTA, and 5 mM QX-314 at pH 7.2 and 288 to 290 mosmol. To isolate EPSC and IPSC cells were first held at −70 and 0 mV, respectively. To isolate monosynaptic responses, all recordings were done in the presence of 1 μM TTX and 100 μM 4AP. Glutamatergic inputs were blocked by bath application of 20 μM NBQX and 50 μM APV.

#### Pharmacological agents

TTX (catalog no. 1078), 4AP (catalog no. 0940), NBQX (catalog no. 0373), and d,l-2-amino-5-phosphonovaleric acid (catalog no. 0106) were obtained from Tocris Bio-Techne; all other salts were obtained from MilliporeSigma.

### In situ hybridization RNA scope

Brains from adult male C57BL/6BJ mice (12 to 15 weeks old) were injected with a retrograde tracing virus carrying eGFP (AAVrg-hSyn-eGFP). After 3 weeks, brains were flash frozen in 2-methyl butane and sectioned at a thickness of 16 μm using a Cryostat (Leica Biosystems). Sections were collected onto Superfrost Plus slides (Daigger Scientific) and subsequently transferred to a −80°C freezer. The *eGFP*, *Hcrtr1*, and *Npffr1* mRNA signals were detected using the RNAscope Hiplex Fluorescent V2 Assay (Advanced Cell Diagnostics) according to the manufacturer’s instructions. Slides were washed twice with washing buffer (2 min each), incubated for 2 min with DAPI and coverslips added using VECTASHIELD (Vector Laboratories). Images were acquired using an ECLIPSE Ti2 inverted microscope (Nikon). Image analysis and cell counting were performed using ImageJ software (Fiji).

### Fiber photometry

The fiber photometry procedure was performed as previously described (*57*). Fluorescent signals were recorded using a commercial Mini Cube Fiber photometry apparatus (Doric Lenses) connected to two continuous sinusoidally modulated LED (DC4100, ThorLabs) tuned at 473 nm (211 Hz) and 405 nm (531 Hz) and two separate photoreceivers (2151, Newport Corporation). Both LEDs were coupled to a large-core (400 μm), high-NA (0.48) optical fiber patch cord, which was mated to a matching brain implant in each mouse. The light intensity at the interface between the fiber tip and the animal ranged from 10 to 20 μW (but was constant throughout each testing session). GCaMP7s and autofluorescence signals were collected by the same fiber and focused onto separate photoreceivers. The data were analyzed by first applying a least-squares linear fit to the 405-nm signal to align it to the 470-nm signal. The resulting fitted 405-nm signal was then used to normalize the 473 nm as follows: Δ*F*/*F* = (473-nm signal − fitted 405-nm signal)/fitted 405-nm signal. Changes in fluorescence following behaviorally relevant events were quantified by measuring the area under the Δ*F*/*F* curve. All experiments were performed in behavioral chambers (Coulbourn Instruments). Behavioral variables, such as shocks and light pulses, were marked in the signaling traces via the real-time processors as TTL signals from the video tracking software.

#### Classification of fluorescence responses

Fluorescence signal recordings were analyzed by segmenting each trial into two distinct periods. A baseline period defined as the first 10 s (0 to 10 s) of the recording and a stimulus period, defined as the subsequent 10 s (10 to 20 s). To quantify baseline fluorescence fluctuations, the mean and SD of fluorescence values were calculated during the baseline period. The peak response to the stimulus was determined by identifying the absolute maximum fluorescence value within the stimulus period while preserving its original sign to distinguish between excitatory and inhibitory responses. Fluorescence responses were classified on the basis of their deviation from baseline variability, using the following criteria: For excitatory responses, the maximum value was positive and exceeded 2 × SD baseline. For the inhibitory responses, the maximum value was negative and was lower than −2 × SD baseline. For no significant change, the maximum value fell within the range of ±2 × SD baseline.

For long-term fiber photometry recording of SCN circadian rhythm, a commercial Mini Cube Fiber photometry apparatus (Doric Lenses), with 465- and 405-nm excitation LEDs, was configured and tethered to animals as describe above. To limit the photobleaching of the fluorescent reporter during the long-term recording, the signal was collected for 4 min every 12 min. Data analysis was performed using a combined methods from work described previously (*49*, *65*). Instead of within sessions, entire 405-nm signal was fitted to 465-nm signal and calculate Δ*F*/*F* = (465-nm signal − fitted 405-nm signal)/fitted 405-nm signal. Then, the second percentile of each trace was taken as the representation of the intracellular calcium level during each session, which represent the level of endogenous circadian rhythm during each session. This intracellular calcium level with 12-min bin was then converted to the file format compatible with ClockLab Analysis for further circadian analysis.

### Two-photon imaging of acute brain slices

Animals were deeply anesthetized using isoflurane and rapidly decapitated. Brain tissue was extracted and immediately immersed in ice-cold, choline-based cutting solution [92 mM choline chloride, 10 mM Hepes, 2.5 mM KCl, 1.25 mM NaH_2_PO_4_, 30 mM NaHCO_3_, 25 mM glucose, 10 mM MgSO_4_, 0.5 mM CaCl_2_, 2 mM thiourea, 5 mM sodium ascorbate, and 3 mM sodium pyruvate (pH 7.4]. Sections (250 to 275 μm thick) were collected using a vibratome (Campden 7000smz-2) and then transferred to a warmed (36°C) choline cutting solution for 10 to 15 min, followed by continued recovery in 36°C oxygenated (95% O_2_/5% CO_2_) ACSF (126 mM NaCl, 21.4 mM NaHCO_3_, 2.5 mM KCl, 1.2 mM NaHPO_4_, 1.2 mM MgSO_4_, 2.4 mM CaCl_2_, and 10 mM glucose) for 30 min. Slices were then kept at room temperature until use. To record, a single slice was transferred to a recording chamber perfused with oxygenated ACSF at room temperature at a flow rate of ~2 ml/min. For imaging, a two-photon microscope (Olympus FVMPE-RS) equipped with an Insight laser was used at an excitation wavelength of 920 nm, and resonant scanning was used at 30 frames per second (fps) with a 20× 0.8-NA water-immersion objective (Nikon). For optogenetic stimulation, a 200-ms-long pulse (20 Hz, 1 ms) was elicited five times using a LED full-field stimulator. Optogentically evoked calcium dynamics were recorded in three conditions: ACSF, 500 nM TTX/100 μM 4-AP, and 10 μM CNQX/ 50 μM D-AP5. Drugs were allowed to perfuse the brain slice for 25 to 30 min before repeating the optogenetic stimulation.

### Two-photon image processing and data analysis

Image registration for two-photon calcium images of brain slices was performed in Fiji (ImageJ). XY rigid-body registration was performed using the Turbo Registration plugin. Cellpose (*77*) was used to segment and identify cell bodies as regions of interest (ROIs), which were exported as masks to use in Fiji.

The change in fluorescence was calculated by generating the average intensity projection of baseline florescence (*F*_0_) during the baseline period. Using the image calculator function in Fiji, *F*_0_ was subtracted from the fluorescent time course *F*, and, then, the resulting image was divided by *F*_0_

$$\text{∆}F/F_{0}=\left(F-F_{0}\right)/F_{0}$$

ROIs generated from Cellpose were then imported into the ROI manager, and the mean intensity of each ROI was measured. The change in fluorescence over the five stimulations was averaged for each cell. Cells were designated excitatory if the change in fluorescence following the stimulation period was greater than 1 SDs from the baseline period. Cells were designated E/I if the change in fluorescence following the stimulation period was greater than 1 SD from the baseline followed by a decrease in signal greater than −1 SD from the baseline. Cells were designated as nonresponders if the change from baseline was not greater than +1/−1 SD.

### Behavioral protocols

#### Locomotor activity rhythms or intracellular calcium in response to footshock, forced swim, or light paired with footshock sessions

For locomotor activity, mice were entrained to a 12-hour L:12-hour D cycle for 7 to 10 days with free access to a stainless-steel running wheel, food, and water. Following this entrainment period, mice were transferred to a constant darkness environment for 6 to 7 days. Subsequently, they were exposed to either a footshock session at CT14, CT22, and CT6, forced swim session, or light paired with footshock session at CT14. After these sessions, mice were allowed to free run for an additional 6 to 7 days. Wheel running activity was continuously recorded in 5-min intervals using VitalView software (Mini Mitter, OR). The total activity and period lengths were calculated using ClockLab (Actimetrics, IL).

##### Footshock session

Mice were placed in an operant chamber and subjected to three unsignaled footshocks (2 s, 0.6 mA) delivered with a 60-s intertrial interval period. This protocol was selected as it was sufficient to induce a significant phase shift at CT14. Mice were habituated for 2 min before footshock presentation, and, at the end of the trial, 1 to 2 min were allowed for recovery before returning to home cage. Control mice were placed in the chamber for the same duration but did not receive any aversive stimuli.

##### Forced swim session

Experiments were performed as previously described (*41*). Each mouse was individually placed into a large 2-liter beaker filled with water maintained at 26°C. Mice were forced to swim for 10 min. Control mice were removed from running wheel bays and placed in a testing area without being subjected to forced swim.

##### Light paired with footshocks

Mice were placed in an operant chamber and exposed to a combined light stimulus and footshock paradigm. After 6 min of continuous light exposure, mice received three unsignaled footshocks (2 s, 0.6 mA) delivered with a 60-s intertrial interval. Mice remained in the chamber for an additional 7 min, resulting in a total light exposure of 15 min. At CT14, a 130-lux (0.4335 W/m^2^) light was used, while, at CT22, a higher intensity of 225 lux (0.6915 W/m^2^) was applied to account for the greater light intensity required to induce phase advances at this circadian time. Light intensities were selected to reliably elicit phase shifts without reaching a ceiling effect. Control mice were placed in the same illuminated chamber for the same duration but did not receive footshocks.

##### Intracellular calcium recordings

For long-term fiber photometry recording of intracellular calcium rhythm, mice were entrained to a 12-hour L:12-hour D cycle for 7 to 10 days before fiber photometry recording. Then, mice were acclimated and entrained to a 12-hour L:12-hour D cycle for an additional 2 days concurrent with long-term fiber photometry recording. After this 2-day acclimation, lights were set to constant darkness. Three footshock (2 s, 0.6 mA, 60-s intertrial interval) or sham control was performed at CT14 of the second day in constant darkness. Calcium signal was then recorded for 3 additional days to monitor phase response to footshocks. Each animal was subjected to a sham (operant chamber with smooth flooring and wallpaper) and footshock session (operant chamber) in a randomized order.

#### Fiber photometry recordings of neuronal responses to footshocks and light

Mice were entrained to a 12-hour L:12-hour D cycle for 7 to 10 days with ad libitum access to food and water. Following entrainment, mice were transferred to constant darkness for 24 hours to eliminate any residual light-mediated neuronal effects. Each mouse was then randomly subjected to three fiber photometry recording sessions at CT6, CT14, and CT22. After habituation to the behavioral chamber (2 min), tethered mice received five unsignaled footshocks (2 s, 1 mA) with a 60-s intertrial interval. This footshock protocol was designed to ensure detection of acute neuronal responses in SCN neurons. After 10 min of the last footshock, mice were exposed to three light pulses 225 lux (0.6915 W/m^2^), each lasting 15 s with a 90-s intertrial interval.

For experiments pairing footshocks and light, mice were placed in operant chamber and a 130-lux light was turned on for CT14 experiments. After 5 min of light on, fiber photometry recordings started with a baseline being recorded for 2 min, and, then, mice were subjected to five presentations of 2-s, 1-mA unsignaled footshock with a 60-s intertrial interval.

For experiments combining chemogenetic manipulation with photometry recordings, NMS-Cre mice received stereotaxic co-injections of AAV9-Flex-jGCaMP7s and AAV8-DIO-hSyn-hM4D(Gi)-mCherry into the SCN region. Two weeks postinjection, mice underwent light entrainment followed by 24 hours in darkness. On the test day, saline or CNO (5 mg/kg, intraperitoneal) was administered at CT13.5 in darkness to induce neuronal inhibition. Thirty minutes later, mice were exposed to a footshock session (2 s, 1 mA) with concurrent photometry recordings.

### Manipulation of PVT neurons

Twelve- to 15-week-old male C57BL/B6J mice were stereotaxically injected with an AAV2-hSyn-hM4D (Gi)-mCherry in the aPVT region. Two weeks postinjection, mice were subjected to locomotor activity rhythms protocol described above. Saline or CNO was intraperitoneally injected (inhibition, 10 mg/kg) at CT13.5. Thirty minutes later, mice were subjected to footshock session.

### Quantification analysis

#### SCN-projecting PVT neurons number

The total area of analysis was manually outlined in coronal brain sections. eGFP^+^ PVT cells were counted using ImageJ. Four representative sections spanning anterior and posterior PVT that contained GFP^+^ cells were selected for analysis. Results obtained from three separate mice were averaged and plotted.

#### PVT inputs to the SCN

Images were converted to 8-bits gray scale, and the optical density (relative to total SCN area) was measured. SCN total area was manually outlined using DAPI as a reference in coronal brain sections. In all cases, five sections comprising rostral, mid, and caudal SCN from four mice were averaged and plotted.

### Statistics and data presentation

All data were plotted and analyzed with GraphPad Prism (version 8.0.1, GraphPad Software). All data are presented as means ± SEM. There were no assumptions or corrections made before data analysis. Differences between two groups were tested with a two-tailed Student’s or two-tailed paired sample *t* test for mice that were retested; differences among multiple groups were examined with ANOVA [one-way and two-way repeated-measures (RM)] followed by Tukey’s test. RM one-way ANOVA was used for repeated measures. For experiments that had missing values, a mixed effect followed by Tukey’s test was used. *P* < 0.05 was considered significant. The sample sizes used in our study, such as the numbers of animals, are typically the same or exceed those estimated by power analysis (power of 0.80, α = 0.05). For behavioral experiments, the sample size is 5 to 17 mice. For fiber photometry analyses, the sample size is three to eight mice. For anatomical and projection analyses, the sample size is three to four mice. For RNAscope experiments the sample size is two mice. For ex vivo Ca^2+^ imaging, the sample size is *n* = 1077 cells, *n* = 1 to 2 slices, and *n* = 2 mice. For electrophysiological recordings of SCN neurons, the sample size is *n* = 21 to 22 cells, *n* = 8 to 12 slices, and *n* = 5 to 7 mice; for drug treatment, the sample size is *n* = 5 to 7 cells, *n* = 5 to 7 slices, and *n* = 5 to 6 mice. All experiments were replicated at least once, and all mice were randomly allocated to the different experimental conditions.

### Manuscript writing and revision

ChatGPT version 4o (OpenAI) was used for grammar and language refinement. Each section of the article was written by the authors and then revised using the following prompts: “revise this paragraph,” “double-check grammar,” and “make this more concise.” All ChatGPT-refined content was reviewed to ensure accuracy and retention of the original meaning.
