# Supplementary material for: A stress-sensing circuit signals to the central pacemaker to reprogram circadian rhythms
Source: Sci Adv. 2025 Jun 20;11(25):eadr7960. doi: 10.1126/sciadv.adr7960 (PMC12180509; doi:10.1126/sciadv.adr7960)
Supplement: Supplementary file 1 — Figs. S1 to S8 Legend for dataset S1 [file sciadv.adr7960_sm.pdf]

Science Advances
AAAS

Supplementary Materials for

A stress-sensing circuit signals to the central pacemaker to reprogram circadian rhythms

Maria E. Yurgel *et al.*

Corresponding author: Maria E. Yurgel, maria.yurgel@nih.gov; Mario A. Penzo, mario.penzo@nih.gov; Samer Hattar, samer.hattar@nih.gov

Sci. Adv. **11**, eadr7960 (2025)
DOI: 10.1126/sciadv.adr7960

The PDF file includes:

Figs. S1 to S8
Legend for dataset S1

Other Supplementary Material for this manuscript includes the following:

Dataset S1

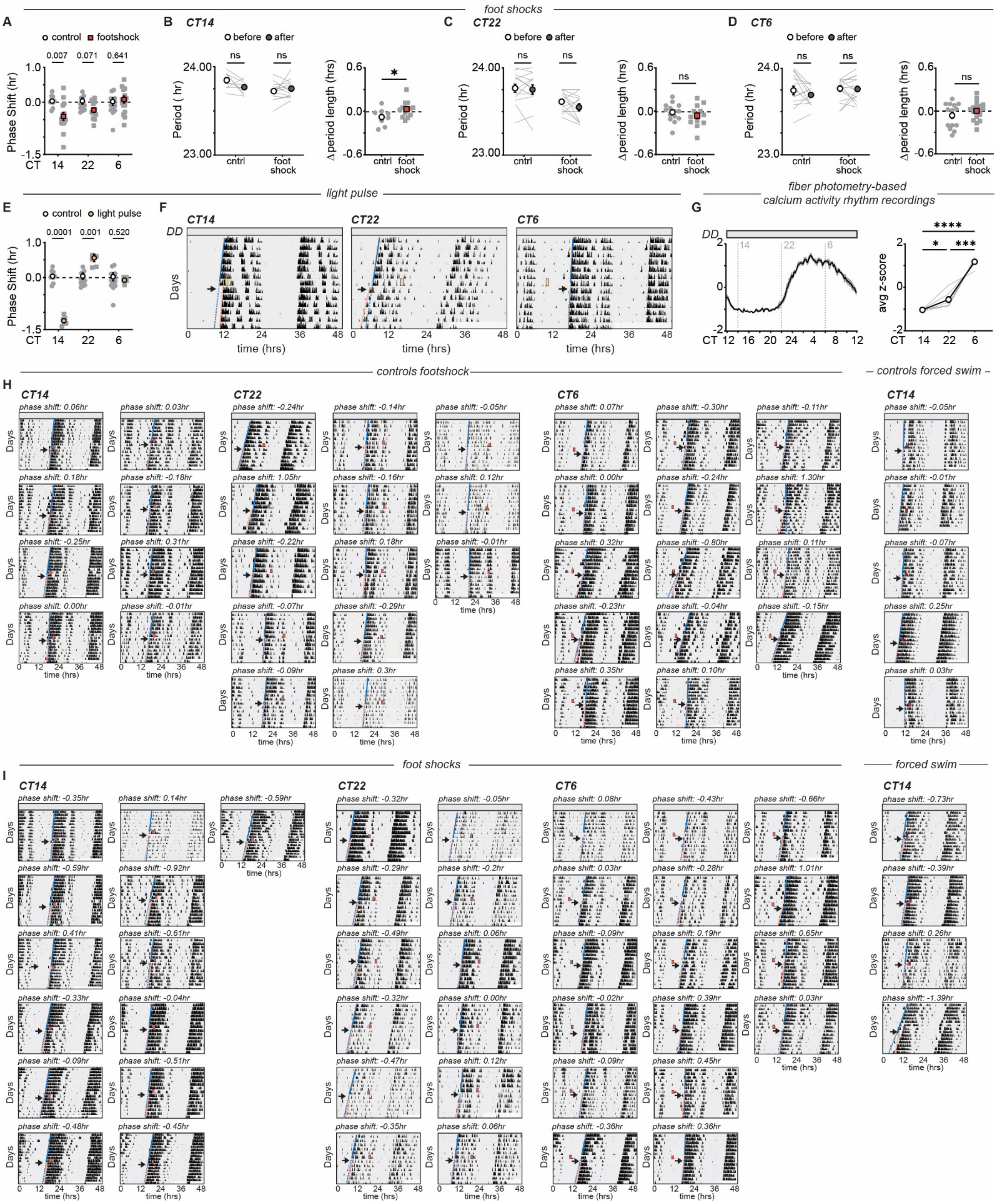
foot shocks
A
control
footshock
0.007
0.071
0.641
Phase Shift (hr)
CT 14 22 6
B CT14
before
after
ns
Period (hr)
cntrl
foot shock
Δperiod length (hrs)
C CT22
Period (hr)
D CT6
light pulse
E
control
light pulse
0.0001
0.001
0.520
F CT14 CT22 CT6
DD
Days
time (hrs)
fiber photometry-based calcium activity rhythm recordings
G
avg z-score
controls footshock
controls forced swim
H CT14
phase shift: 0.06hr
phase shift: 0.03hr
phase shift: 0.18hr
phase shift: -0.18hr
phase shift: -0.25hr
phase shift: 0.31hr
phase shift: 0.00hr
phase shift: -0.01hr
CT22
phase shift: -0.24hr
phase shift: -0.14hr
phase shift: -0.05hr
phase shift: 1.05hr
phase shift: -0.16hr
phase shift: 0.12hr
phase shift: -0.22hr
phase shift: 0.18hr
phase shift: -0.01hr
phase shift: -0.07hr
phase shift: -0.29hr
phase shift: -0.09hr
phase shift: 0.3hr
CT6
phase shift: 0.07hr
phase shift: -0.30hr
phase shift: -0.11hr
phase shift: 0.00hr
phase shift: -0.24hr
phase shift: 1.30hr
phase shift: 0.32hr
phase shift: -0.80hr
phase shift: 0.11hr
phase shift: -0.23hr
phase shift: -0.04hr
phase shift: -0.15hr
phase shift: 0.35hr
phase shift: 0.10hr
CT14
phase shift: -0.05hr
phase shift: -0.01hr
phase shift: -0.07hr
phase shift: 0.25hr
phase shift: 0.03hr
I
foot shocks
forced swim
CT14
phase shift: -0.35hr
phase shift: 0.14hr
phase shift: -0.59hr
phase shift: -0.59hr
phase shift: -0.92hr
phase shift: 0.41hr
phase shift: -0.61hr
phase shift: -0.33hr
phase shift: -0.04hr
phase shift: -0.09hr
phase shift: -0.51hr
phase shift: -0.48hr
phase shift: -0.45hr
CT22
phase shift: -0.32hr
phase shift: -0.05hr
phase shift: -0.29hr
phase shift: -0.2hr
phase shift: -0.49hr
phase shift: 0.06hr
phase shift: -0.32hr
phase shift: 0.00hr
phase shift: -0.47hr
phase shift: 0.12hr
phase shift: -0.35hr
phase shift: 0.06hr
CT6
phase shift: 0.08hr
phase shift: -0.43hr
phase shift: -0.66hr
phase shift: 0.03hr
phase shift: -0.28hr
phase shift: 1.01hr
phase shift: -0.09hr
phase shift: 0.19hr
phase shift: 0.65hr
phase shift: -0.02hr
phase shift: 0.39hr
phase shift: 0.03hr
phase shift: -0.09hr
phase shift: 0.45hr
phase shift: -0.36hr
phase shift: 0.36hr
CT14
phase shift: -0.73hr
phase shift: -0.39hr
phase shift: 0.26hr
phase shift: -1.39hr

Fig. S1. Stressors phase shift circadian rhythms differently than light

(**A**) Comparison between control and footshock groups across CT times. (CT14, *n*=9 to 14; CT22, *n*=13 to 14; CT6, *n*=15 to 17 mice. *p-values* from two-way ANOVA with Sidak's test are indicated on the graph. (**B** to **D**) Quantification of circadian period (hr) before (white) and after (gray) treatment in control mice placed in operant chamber or mice subjected to a session of footshock at CT14 (B, *n* = 9 to 14 mice), 22 (C, *n* = 13 to 14 mice), and 6 (*n* = 15 to 17 mice). Not significant, two-way ANOVA, Sidak's test (left). Quantification of change in period length in control and footshock treated mice at CT14. *P = 0.030, student's t-test (right). (**E**) Comparison between control (white) and light pulse groups (yellow) across CT times. (CT14, *n*=9 and 6; CT22, *n*=14 and 6; CT6, *n*=15 and 6 mice. *p-values* from two-way ANOVA with Sidak's test are indicated on the graph. **(F)**Wheel running activity profiles of mice subjected a light pulse at CT14, 22 and 6. **(G)** Average changes in fluorescence (z-score) across 24 hours in constant dark (left). Average z-score per hour at CT14, 22, and 6. (*n*= 7 mice). *P = 0.024, ***P = 0.0005, ****P < 0.0001. RM one-way ANOVA. **(H** and **I)** Wheel running activity profiles across CT times and corresponding magnitude of phase shifts in control mice (H) and mice subjected to a footshocks and forced swim sessions (I). Rectangles represent day and time of test. Blue solid line depicts activity onset prior to test; blue dotted line depicts expected activity onsets, and pink line, depicts shift in activity onset post-test. Black arrow indicated the first day post-test. Data are means ± SEM.

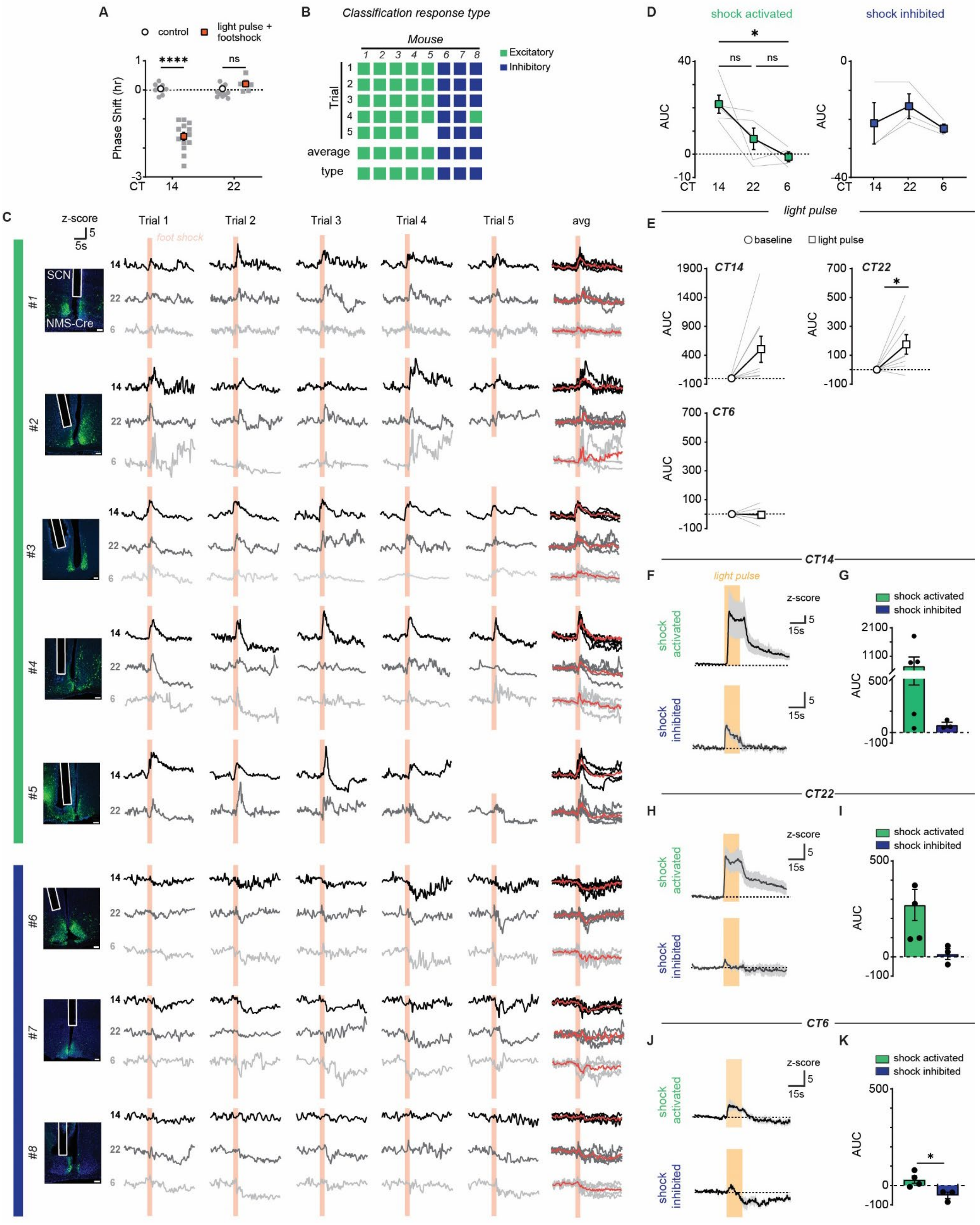
A
control
light pulse + footshock
****
ns
Phase Shift (hr)
CT
14
22
B
Classification response type
Mouse
Trial
average
type
Excitatory
Inhibitory
C
z-score
5s
Trial 1
Trial 2
Trial 3
Trial 4
Trial 5
avg
foot shock
SCN
NMS-Cre
#1
#2
#3
#4
#5
#6
#7
#8
D
shock activated
shock inhibited
AUC
light pulse
E
baseline
light pulse
CT14
CT22
CT6
F
light pulse
shock activated
shock inhibited
15s
G
shock activated
shock inhibited
H
I
J
K

Fig. S2. SCN^{NMS+} neurons are differentially responsive to light and aversive stimuli across circadian times

(**A**) Comparison of phase shifts (hr) between control and light paired with footshock groups across CT times. (CT14, *n*=9 and 13; CT22, *n*=6 to 12 mice. ****$P < 0.0001$ and ns=0.369. two-way ANOVA, Sidak's test **(B)** Classification of response type (shock activated or shock inhibited) based on CT14 responses across trials per mouse for GCaMP7s photometry experiments shown in Figure 2. (**C**) Optical fiber placement for shock-activated (green bar) and shock-inhibited (blue bar) groups, and individual GCaMP7s trials and average response to footshocks across CT times. Scale bar: 100µm. **(D)** Area under the curve (AUC) comparison in shock activated (*n* = 4 to 5 mice, green) and shock inhibited (*n* = 3 mice, blue) groups across CT times. *$P = 0.031$, shock activated, Mixed-effects one way ANOVA, Tukey's test; shock inhibited, RM one way ANOVA, Tukey's test **(E)** Comparison of AUC between baseline and light pulse conditions at CT14, 22, and CT6. (*n* = 7 to 8 mice). *$P = 0.036$, two-tailed paired t-test. **(G, H and J**) Average GCaMP7s responses to light in mice classified as shock-activated or shock-inhibited at CT14 (G), 22 (H), and 6 (J). Note scale change for shock activated group at CT14. Light pulse duration (15s) is depicted by yellow shaded area. (**G, I, and K)** AUC comparison of light responses between shock activated and shock inhibited groups at CT14 (G), CT22 (I), CT6 **(**K)**.** CT14, shock activated, (*n* = 5 mice), shock inhibited, (*n* = 3 mice). CT22, shock activated, (*n*=5 mice), shock inhibited (*n* = 3 mice). CT6, shock activated, (*n* = 4 mice), shock inhibited, (*n* = 3 mice), *$P = 0.027$. Student's *t*-test. Data are means ± SEM.

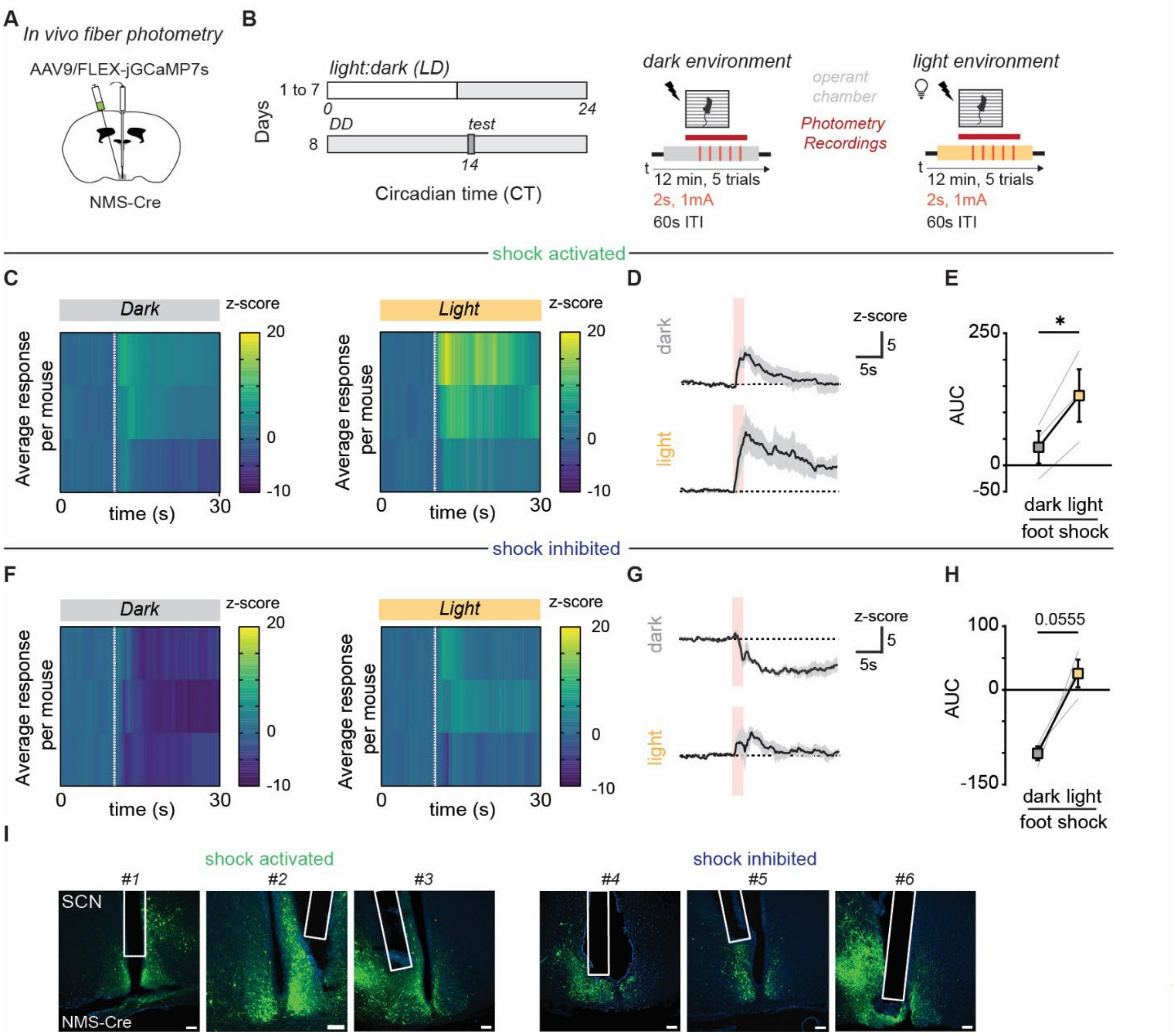

Fig. S3. Pairing light and stressors disinhibits SCN microcircuit at CT14

(A) Schematic of the viral vector strategy for fiber photometry imaging of SCN^{NMS+} neurons. (**B**) Schematic of experimental paradigm; mice were entrained to 12L:12D cycle, then placed in constant dark (DD) for 24hrs and tested at CT14 (left). Schematic of footshock session in a dark or light environment. (**C** and **F**) Heat map showing the mean GCaMP7s responses per mouse in SCN^{NMS+} neurons, averaged over five footshock trials, in mice displaying increases (shock activated, C) or decreases in fluorescence levels (shock inhibited, F) in a dark or light environment. (**D** and **G**) Average GCaMP7s response from SCN^{NMS+} neurons in response to footshocks (2s, shaded area) in dark or light environments in the shock activated (D) and shock inhibited groups (G). ($n = 3$ mice). (**E** and **H**) Quantification of GCaMP7s responses to footshocks from SCN^{NMS+} neurons for mice exposed to a dark or light environment in shock activated (E) and inhibited groups (H). Data are means ± SEM. (n=3 mice). Shock activated, *P =0.045. Shock inhibited, $P = 0.055$. Two-tailed paired-sample t-test. (**I**) Optical fiber placement (SCN) for shock-activated and shock-inhibited groups. Scale bar: 100μm.

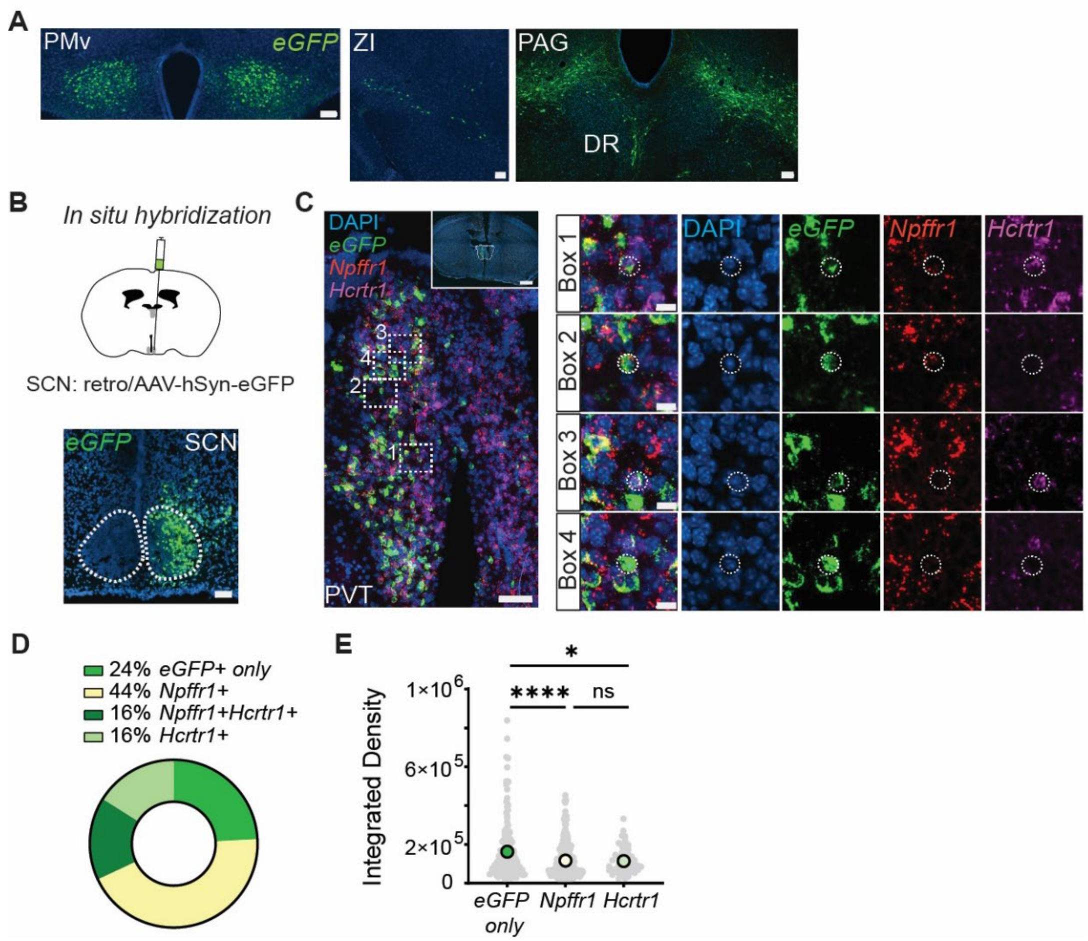

Fig. S4. Distinct subpopulations of PVT neurons project to the central pacemaker

(**A**) Representative images of the distribution of retrogradely labelled cells found in the ventral premammillary nucleus (PMv), zona incerta (ZI), periaqueductal of gray (PAG), dorsal raphe (DR). Scale bar:100µm. (**B**) Schematic of the retrograde viral strategy used to label SCN-projecting PVT neurons combined with *in-situ* hybridization to molecularly characterize SCN projectors (top). Representative image showing injection site in the SCN (bottom). Scale bar: 100µm. (**C**) Representative zoomed out image of *eGFP* expression in the PVT (dotted white line, inset). Scale bar: 1mm. Representative image of *in situ* labeling *eGFP*, *Npffr1*, and *Hcrtr1* in the PVT. Numbered rectangles show the location of SCN projectors (eGFP+) depicted on the right. Zoomed in images of SCN projectors (eGFP+, right). Box 1 shows SCN-projector expressing *Npffr1* and *Hcrtr1*. Box 2 shows an SCN projector expressing *Npffr1* only. Box 3 shows an SCN projector expressing *Hcrtr1* only. Box 4 shows an SCN projector that neither express *Npffr1* nor *Hcrtr1*. Scale bar: 100µm. (**D**) Pie chart illustrating the molecular composition of *eGFP*+ PVT neurons projecting to the SCN. Green represents neurons expressing only *eGFP*, yellow represents neurons expressing only *Npffr1*, dark green indicates neurons co-expressing *Npffr1* and *Hcrtr1*, and light green shows neurons expressing only *Hcrtr1*. (**E**) Integrated density measuring mRNA expression in *eGFP*+ only (n = 156 cells). *Npffr1*+ (n = 300 cells). *Hcrtr1*+ (n = 48 cells). Data are means ± SEM. (n = 2 mice). *P = 0.0184, ****P < 0.0001, one-way ANOVA, Tukey's test

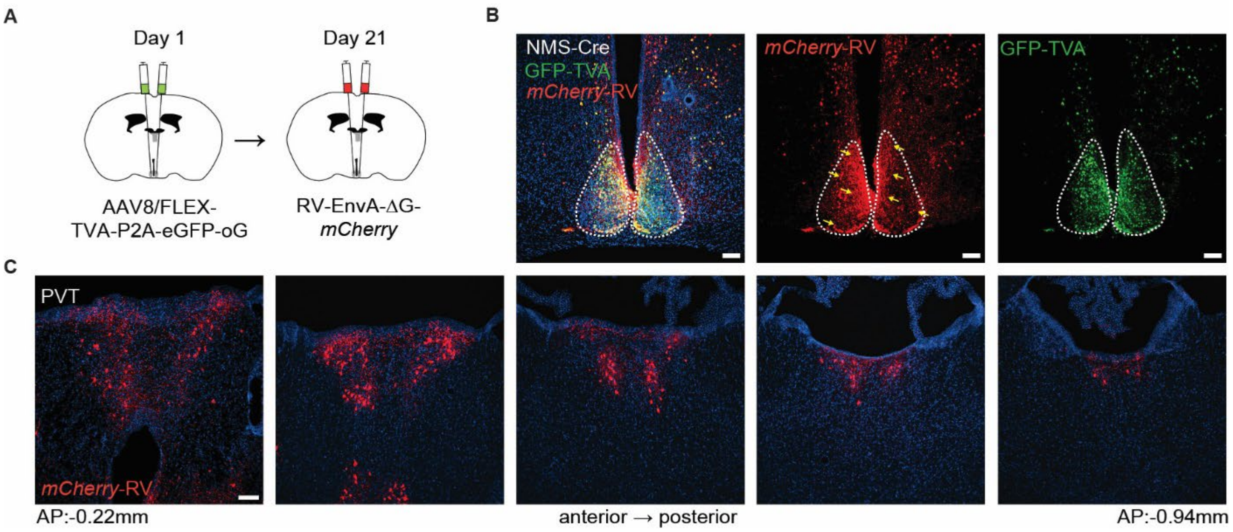

Fig. S5. PVT neurons project to SCN^{NMS+} neurons

(**A**) Schematic of the monosynaptic rabies virus strategy used to label PVT neurons projecting specifically to SCN^{NMS+} neurons. (**B**) Representative image depicting injection site. SCN^{NMS+} neurons express GFP-TVA (green) and mCherry-RV (red). Yellow arrows depict fibers innervating the SCN. Scale bar: 100µm. **(C**) Representative coronal sections showing labeling of monosynaptic inputs from the PVT to SCN^{NMS+} neurons. Scale bar: 100µm.

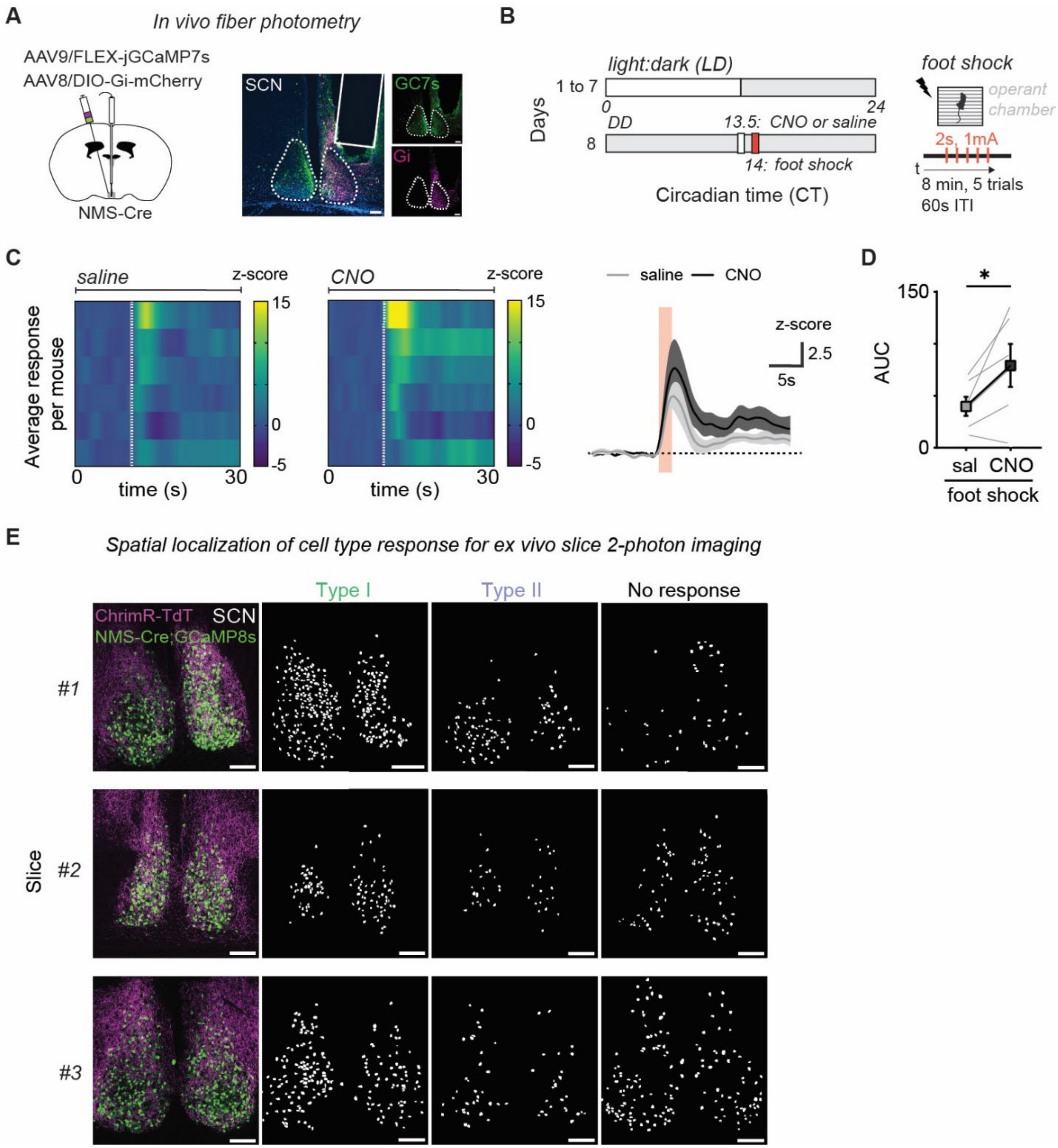

Fig. S6. Silencing local inhibitory SCN microcircuits enhances SCN^{NMS+} neuronal response to footshocks

(**A**) Schematic of the dual virus strategy used to both record from and silence SCN^{NMS+} neurons. Representative images showing the injection site and expression pattern for GCaMP7s and DIO-Gi-mCherry in the SCN. Scale bar: 100µm (**B**) Schematic of experimental paradigm (**C**) Heat map showing average GCaMP7s responses per mouse across five footshock trials in saline- and CNO-injected groups. Line graph depicts mean GCaMP7s response to 2 s footshocks (shaded area) in each group (n = 6). (**D**) Comparison of area under the curve (AUC, 10–30 s) between

saline- and CNO-injected mice. Data are means ± SEM. *P =0.043. Two tailed paired t-test. (**E**) Image showing aPVT-originating fibers (magenta) and GCaMP8s expression in the SCN (green) of NMS-Cre mice, captured from slices used in *ex vivo* 2-photon calcium imaging. Black and white images display the distribution of SCN neurons categorized by their response types: Type I (green, net increases), Type II (purple, increases followed by decreases), and non-responsive neurons (black). Scale bar 100µm.

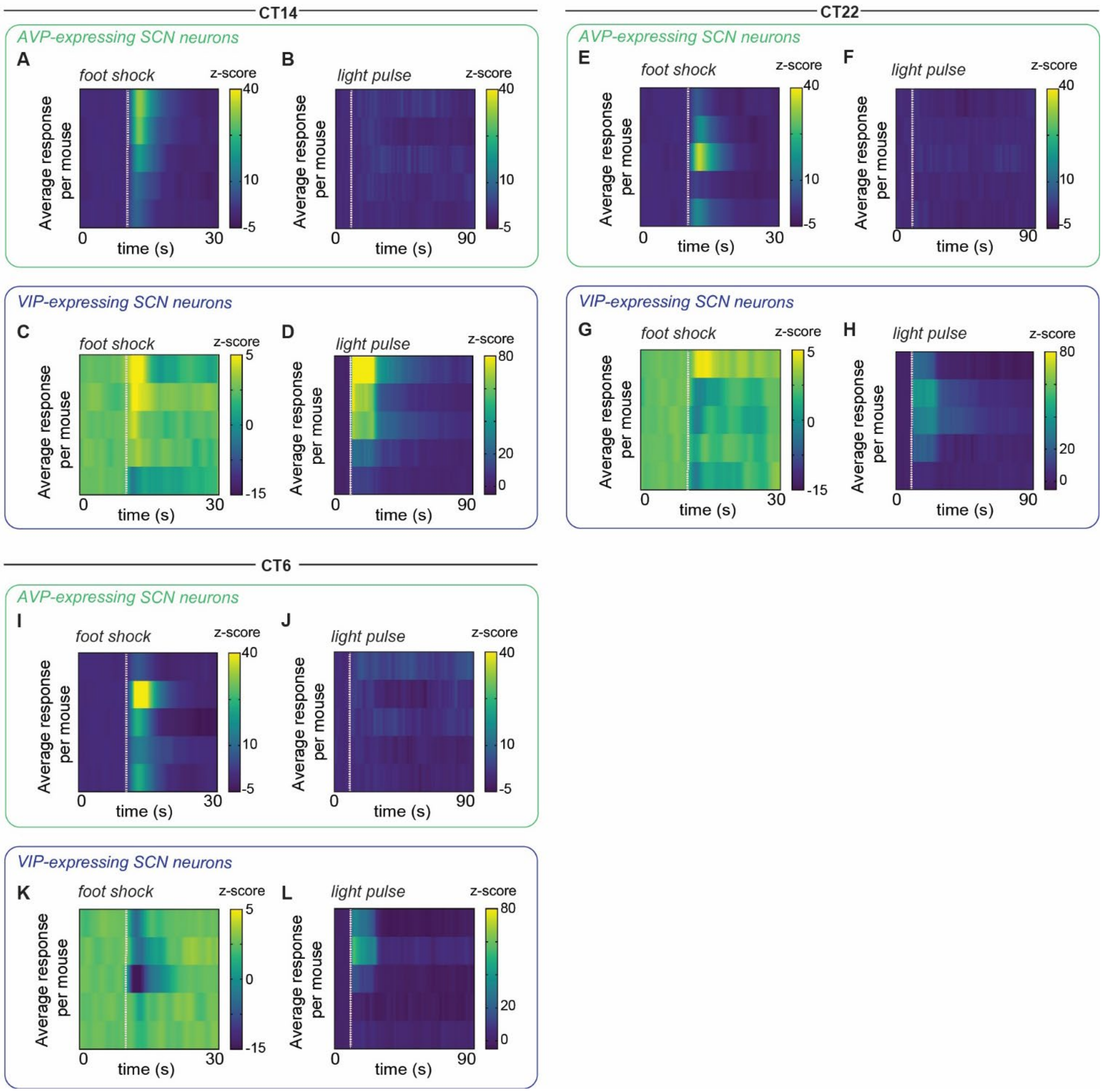

Fig. S7. The SCN differentially encodes stress information in SCN^{AVP+} and SCN^{VIP+} neurons

Heat maps showing average GCaMP7s responses in SCN^{AVP+} and SCN^{VIP+} neurons to footshock (5 trials/mouse) and light stimuli (3 trials/mouse) at different circadian times (CT). (**A and B**) SCN^{AVP+} responses to footshock (A) and light (B) at CT14. (**C and D**) SCN^{VIP+} responses to footshock (C) and light (D) at CT14. (**E–F**) SCN^{AVP+} responses to footshock (E) and light (F) at CT22. (**G and H**) SCN^{VIP+} responses to footshock (G) and light (H) at CT22. (**I and J**) SCN^{AVP+} responses to footshock (I) and light (J) at CT6. (**K and L**) SCN^{VIP+} responses to footshock (K) and light (L) at CT6. Footshock and light pulse administration are indicated by white dotted line.

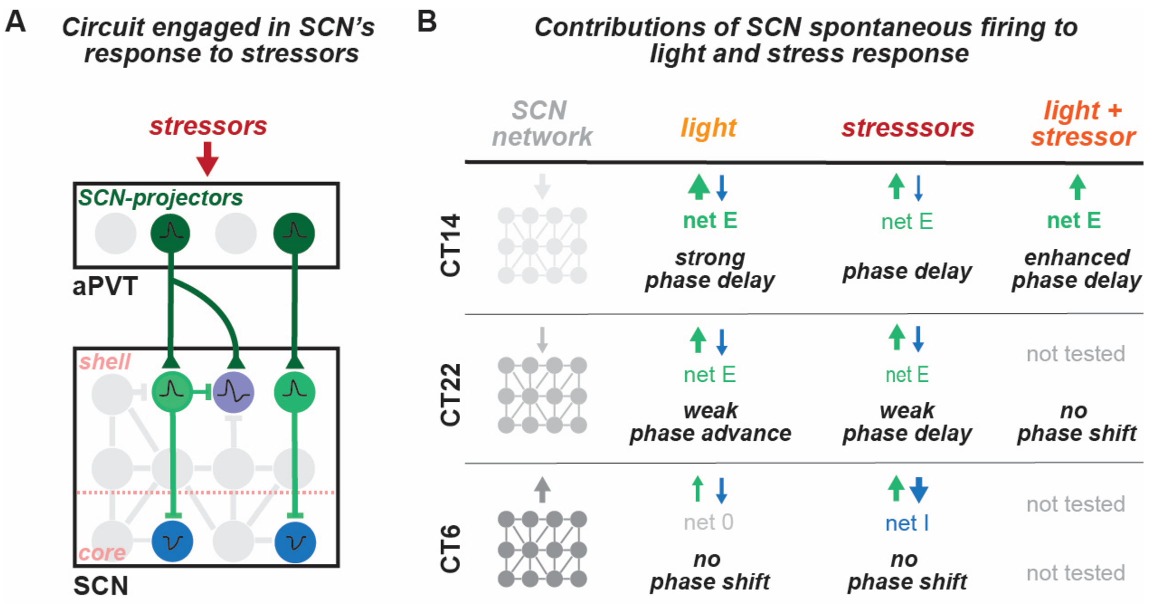

Fig. S8. Proposed model of circuits for SCN's response to environmental cues

(A) Stressors, such as footshock, activate a subset of aPVT neurons that project to the SCN (dark green). Dense excitatory inputs from the aPVT innervate SCN^{NMS+} neurons in the SCN shell (light green), which express AVP. This activation can induce feedforward inhibition (FFI) in many neurons, leading to biphasic responses (purple). The recruitment of SCN FFI extends to the core, which lacks dense excitatory input from the PVT. This likely occurs through AVP-expressing neurons providing inhibitory input to VIP-expressing neurons (blue) in the SCN core, resulting in suppression of VIP neurons to footshocks. Additionally, the intrinsic inhibitory tone of the SCN local network (light gray), driven by spontaneous SCN activity, further shapes SCN neuronal responses to stressors. **(B)** SCN neurons exhibit varying levels of spontaneous activity across the circadian cycle. During the early night (CT14, light gray), SCN neurons are silent. In the late night (CT22, gray), spontaneous activity increases slightly, and during the day (CT6, dark gray), SCN neurons are highly active. We propose that these daily changes in SCN spontaneous activity modulate the excitatory/inhibitory (E/I) balance of the SCN's response to external stimuli, thereby influencing the magnitude of phase shifts. At CT14, when the SCN network is silent, external stimuli, such as light and stressors, elicit a net excitatory response (net E). This net E response promotes phase delays, with higher excitatory imbalance resulting in larger phase shifts. Pairing light and stressors reduces inhibition further enhancing the phase shifts. At CT22, when the SCN network shows moderate spontaneous activity, the net response to stimuli remains positive but is weaker than at CT14, leading to smaller phase shifts. Pairing light and stressors does not induce phase shift, likely due to the response of overlapping yet functionally distinct SCN microcircuits engaged at CT22 compared to CT14. At CT6, when SCN neurons are highly active, SCN neuronal activity becomes more balanced (net 0) or biased toward inhibition (net I), resulting in no phase shifts. Green and blue arrows indicate activation and inhibition derived from the responses of SCN^{AVP+} and SCN^{VIP+} neurons to light and footshocks across circadian times. The combined activity of SCN subpopulations shapes the net excitatory (E), inhibitory (I), or neutral (zero) response observed in SCN^{NMS+} neurons.

Dataset S1: Supplementary Excel file. Raw data corresponding to all main and supplementary figures. Each tab contains data associated with specific figure panels as labeled.
